# Mutual gaze and movement synchrony boost observers’ enjoyment and perception of togetherness when watching dance duets

**DOI:** 10.1038/s41598-024-72659-7

**Published:** 2024-10-14

**Authors:** Emily S. Cross, Kohinoor M. Darda, Ryssa Moffat, Lina Muñoz, Stacey Humphries, Louise P. Kirsch

**Affiliations:** 1 Professorship for Social Brain Sciences, ETH Zürich, Zurich, Switzerland; 2 ARISA (Advancement and Research in the Sciences and Arts) Foundation, Pune, India; 3 Goldsmiths, University of London, London, UK; 4 Integrative Neuroscience and Cognition Center, UMR 8002, CNRS, Université Paris Cité, Paris, France

**Keywords:** Human behaviour, Perception, Cooperation

## Abstract

As social beings, we are adept at coordinating our body movements and gaze with others. Often, when coordinating with another person, we orient ourselves to face them, as mutual gaze provides valuable cues pertaining to attention and intentions. Moreover, movement synchrony and mutual gaze are associated with prosocial outcomes, yet the perceptual consequences of these forms of coordination remain poorly understood. Across two experiments, we assessed how movement synchrony and gaze direction influence observers’ perceptions of dyads. Observers’ behavioural responses indicated that dyads are perceived as more socially connected and are more enjoyable to watch when moving synchronously and facing each other. Neuroimaging results showed modulation of the Action Observation and Theory of Mind networks by movement synchrony and mutual gaze, with more robust brain activity when evaluating togetherness (i.e., active and intentional collaboration) than aesthetic value (i.e., enjoyment). A fuller understanding of the consequences of movement synchrony and mutual gaze from the observer’s viewpoint holds important implications for social perception, in terms of how observers intuit social relationships within dyads, and the aesthetic value derived from watching individuals moving in these ways.

## Introduction

From step dancing to square dancing, from ballrooms to TikTok takes, humans have long watched and performed partner dances. Key features in these examples, and many other dance traditions as well, include dancers making synchronised movements and looking directly at each other. Theories regarding the origins and functions of human dance have emerged from disciplines as diverse as anthropology, sociology, biology, and neuroscience. One particularly compelling and recent idea is that the evolutionary purpose of dance could be to instantiate interpersonal coordination through imitation and synchrony^1,2^. In line with this, the fields of behavioural psychology and cognitive neuroscience offer a wealth of evidence confirming that engaging in synchronised movements and mutual gaze–features of many dance forms–can indeed bolster interpersonal coordination, as well as the establishment and strengthening of social bonds. However, the perspective of the observer has often been overlooked, and clearly just as many (if not more) people regularly engage in watching dance as dancing themselves, and often derive pleasure from doing so. How do observers “read” the social dynamics between two movers, whether dancing or in everyday life, based on movement synchrony and mutual gaze? Furthermore, how might these factors impact aesthetic appraisals (defined here as the amount of enjoyment an observer obtains from watching human movement)? Here we present findings from one behavioural and one neuroimaging experiment with the aim of addressing these questions from neural and behavioural perspectives.

### Mechanisms and perceptual consequences of movement synchrony

Movement synchrony, also called motor synchrony, emerges when two or more individuals match their movements in time and space^3,4^. An established literature documents how both spontaneous and induced synchrony can lead to increased prosocial behaviours, enhanced feelings of affiliation^5^, liking^6^, rapport^7^ and closeness toward one’s synchronised counterpart^4,8,9^. Beyond the socially relevant benefits for synchronised people, observers of synchronous movements experience greater enjoyment as the degree of synchrony increases^10,11^. Recent work by Tang Poy and Woolhouse^12^ explored the relationship between synchrony between two hip-hop dancers, and synchrony between the dancers and music, and found that aesthetic evaluations were more strongly driven by synchrony between the dancers than the dancers synchronising with the music. Moreover, observers who watch dyads moving in synchrony not only perceive those dyads to share more rapport and social “togetherness” (defined as belonging to a social unit with joint agency), but these judgements are further based on psychological inferences that extend beyond simple perceptual features^13–15^.

At the neural level, movement synchrony has mainly been considered in the context of what happens when we physically engage in imitation. This research has put substantial emphasis on the role of the frontoparietal cortices of the Action Observation Network (AON)^16^. The AON includes the inferior frontal gyri (IFG), inferior parietal lobules (IPL), premotor cortices (PMC) and superior temporal sulci (STS). Recently, Shamay-Tsoory and colleagues^17^ put forth a more detailed theoretical account of the observation, execution, and reward-based mechanisms that could underpin maintenance of movement synchrony, implicating a broader neural network. This theory, called the extended integrative model of alignment, suggests that the prefrontal and anterior cingulate cortices, as well as anterior insulae, monitor and compare predictions about upcoming movements to the real outcomes, serving to spot gaps in alignment. When a mismatch is detected between one’s own and an observed movement, the canonical AON regions (IFG, IPL, PMC) facilitate greater alignment–or synchrony–by upregulating action observation-execution coupling. In the case that alignment is strong and no gaps are detected, a subnetwork of reward-related brain areas are recruited (orbitofrontal cortices, ventromedial prefrontal cortex [vmPFC], and ventral striatum). This model explains how our brains help us stay aligned, or in sync, with one another, and highlights the roles of observation, execution, and reward processes.

In contrast to the neural correlates of engaging in synchrony, the neural correlates of *observing* synchronous movements have received less attention, despite the ubiquity of synchronous actions we perceive in daily life^18^. While observing synchrony, one does not need to monitor one’s own body movements. Nonetheless, one may simulate the observed body movements made by others, thereby engaging the sensorimotor regions recruited for monitoring gaps when moving in synchrony^19–22^. This is, in fact, suggested in one of the few studies presenting relevant data, conducted by Georgescu and colleagues^18^. For this study, the authors implemented perfectly synchronous movements as the control condition to examine how temporally contingent (vs. mirrored) movements engage brain networks associated with social and action perception. While the focus of this study was on the influence of temporal and social contingency of movements (i.e., coherence) and movement smoothness^18^, we are particularly interested in their findings pertaining to synchronous movements. They employed a 2 × 2 factorial design wherein participants watched facing pairs of computer animated mannequin models move smoothly or rigidly, in either a temporally contingent or mirrored (i.e., synchronous) way. Georgescu et al. demonstrated that core regions of the AON, including the left IPL, right posterior STS and right IFG, responded more robustly to temporally contingent movements than synchronised mirrored movements. The authors interpreted this as evidence that the AON is preferentially tuned to perceive temporally contingent over synchronised/mirrored social encounters between two individuals^18^. We aim to complement Georgescu et al.’s work by comparing synchronised movements to asynchronous movements in a context where both are equally ecologically valid, thereby offering further insight into the AON’s allegedly low sensitivity to synchrony.

Georgescu et al.^18^ also observed that the bilateral dorsal medial prefrontal cortex (dmPFC) and left temporoparietal junction (TPJ) did not show any sensitivity to temporal contingency or movement synchrony in movements but did show sensitivity to the rigid, more than the smooth, movements. These brain regions are typically associated with forms of social processing, such as inferring others’ thoughts, goals, or intentions, cognitive processes commonly referred to as theory of mind (ToM). The authors interpret this to mean that these regions are sensitive to the mental conflict that emerges when observers expect human figures to move in a smooth, biological way, and these expectations are then violated by a different movement pattern. Other researchers have demonstrated that portions of the AON might be similarly sensitive to form and motion mismatches^23^. In the present study, by comparing neural responses to synchronous and asynchronous movements, we aim to enrich our understanding of the AON and ToM network’s contributions to social information processing with evidence from intentionally synchronised movements performed by real people.

One further piece of empirical evidence informing our understanding of perceived synchrony comes from a study by Moffat and Cross, who explored the cortical underpinnings of enjoyment of synchronous movements^24^. In this behavioural and functional near infrared spectroscopy (fNIRS) study, participants mirrored a stick figure’s upper body movements, then viewed videos of pairs of stick figures playing the mirror game (i.e., copying each other’s movements with the aim of achieving synchrony). Participants rated their enjoyment of the synchronous movements they watched, as well as whether they recognised the movement sequences as those they had mirrored themselves. The authors found that ratings of enjoyment were greatest for movement sequences that participants had mirrored, particularly when participants recognised having experienced the movements with their own bodies. Greater enjoyment of movement sequences was associated with increased activation solely of the left superior temporal gyrus (STG), while increased recognition of having mirrored a movement sequence was associated with increased activation of the left IFG, bilateral STG, and right IPL (regions of the AON). Moffat and Cross interpret the involvement of canonical AON regions, more so for recognition than enjoyment of synchrony, as evidence that recognising movements draws on more cognitive resources related to movement prediction than enjoyment of movements^24^. This first foray combining aesthetic ratings and brain activity offers initial insight into the neural underpinnings of synchrony appreciation, but did not consider gaze information. As we detail below, dyads’ gaze direction is another crucial cue that shapes social perception.

### Mechanisms and social consequence of gaze direction

Like movement synchrony, engaging in mutual gaze with another person also has well-documented prosocial consequences on those actively involved^25–27^. Studies demonstrate that mutual gaze is important in building trust and social bonding between human infants and caretakers^28^, and is further associated with the release of oxytocin, a natural hormone that plays a pivotal role in establishing and maintaining filial attachments throughout the lifespan^29,30^.

Compared to movement synchrony, a more developed empirical evidence base contributes to our knowledge about the neural mechanisms and behavioural consequences of perceiving other people engage in mutual gaze. This is due to the rich, informative nature of mutual gaze as a social cue for signifying perceived events as social interactions. Work by Koldewyn and colleagues documents how parts of the occipitotemporal cortex (in particular, a portion of the posterior STS) are sensitively tuned to detect social interactions between two individuals^31–33^. These authors have proposed that interactive dyads are thus processed in the brain as more than the sum of their parts. Moreover, work by Abassi and Papeo explored the perception of faces and bodies that were face-to-face and back-to-back^34–36^. These authors report that superior portions of the lateral occipital cortex respond more robustly when viewing two bodies that are facing toward each other^35^, while another part of this same cortical region appears to generalise across body and face direction cues, possibly integrating signals from category-specific (i.e., face and rest-of-the-body) visual areas to form a representation of the social relationship between dyads^34^.

### Current study

Although the prosocial effects of moving together and mutual gaze have been well established in those moving or interacting, we still have considerable gaps in our knowledge about how these factors interact during social perception. In addition, while several published studies inform our understanding of the perception of social cohesiveness among dyads moving together, we know that many of the world’s dance traditions feature such dyads, but the influence of movement features and gaze directions on the aesthetic value of such dance duets remains unexplored. Therefore, across one behavioural and one brain imaging experiment, here we examined critical questions pertaining to the role of gaze direction and synchrony in judgements of aesthetic value (i.e., enjoyment) and togetherness (i.e., joint agency).

**Fig. 1:**
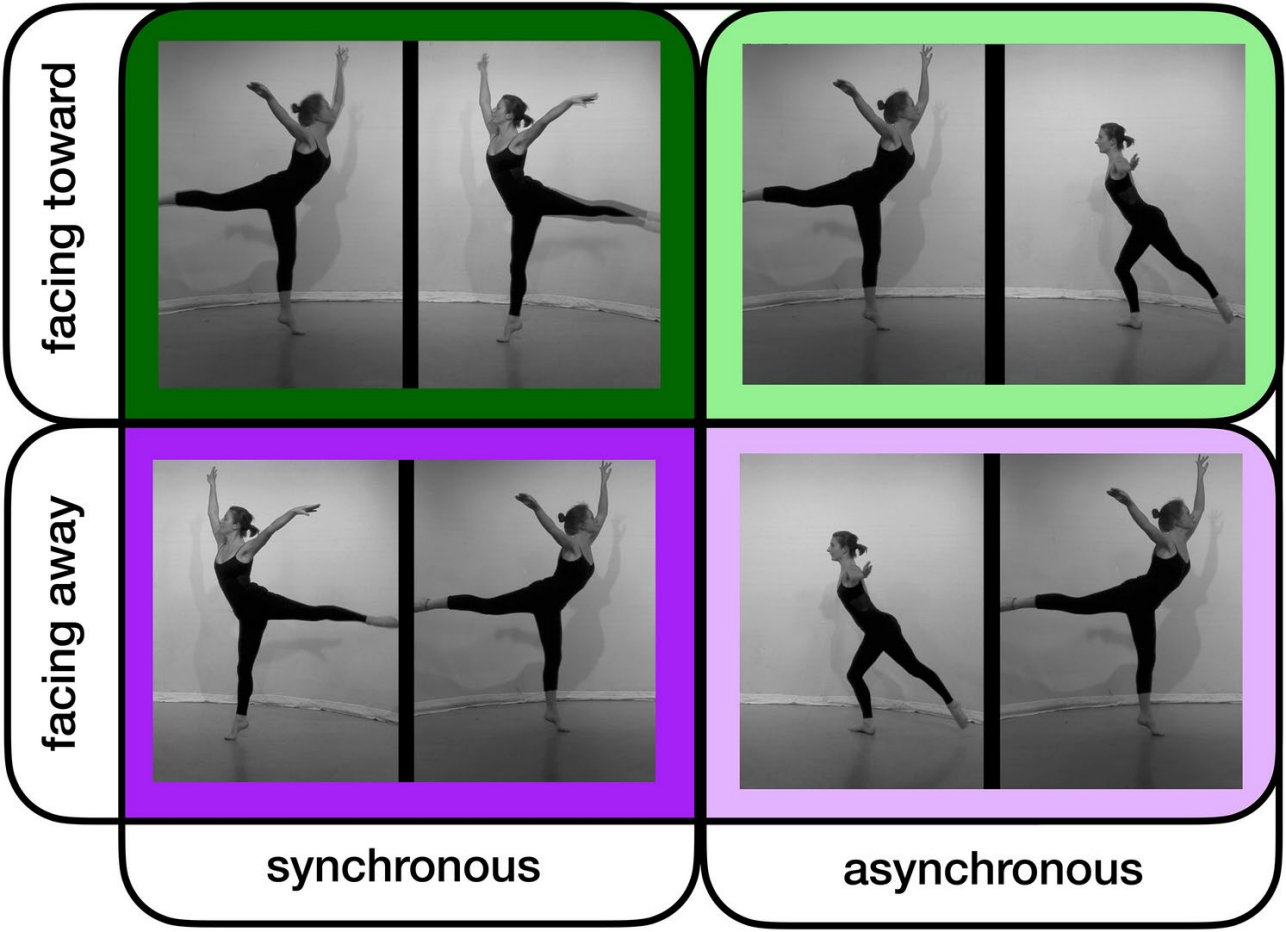
A 2 (movement kinematics: synchronous vs. asynchronous movements) × 2 (gaze direction: facing toward vs. facing away) factorial design was employed, leading to four different conditions (note: background colours for each design cell depicted here correspond to data plotted in Fig. 4).

First, consistent with recent suggestions to make replication a foundational practice in psychology^37^, we sought to replicate the effect of synchrony and gaze direction on the perception of togetherness as measured by behavioural ratings. We predicted that for both aesthetic judgements and togetherness evaluations, participants would assign the highest values when dyads moved synchronously and faced each other, whereas ratings would be lowest when dyads moved asynchronously and faced away from each other^12,14^. Next, we used functional magnetic resonance imaging (fMRI) to investigate the extent to which responses in brain regions associated with networks engaged in social processing (ToM) and action observation (AON) are modulated by gaze direction and movement synchrony (see Figure 1), as well as the extent to which such responses are further modulated by ratings of togetherness and aesthetic judgement. Contrary to Georgescu and colleagues^18^ (though see above for reasons why), and in keeping with recent findings from Moffat and Cross^24^, we predicted that AON regions would respond more robustly when dyads move synchronously. Given the importance of facing direction for inferring social relationships among observed dyads^31–33^, we predicted the most robust engagement of the ToM network would emerge when the dyads faced each other. For hypotheses concerning the role played by the AON when making aesthetic appraisals, here we might expect to see modulation of parietal^38^ and premotor cortices^39^ with increasing enjoyment, with the prediction that mutual gaze and synchrony should lead to the highest enjoyment ratings^12^. While the role of the ToM brain network has not yet been firmly established in aesthetic processing^40^, the notion that perspective taking, belief attribution, imagination, and other cognitive processes relevant to theory of mind contribute to aesthetic processing is well established^41–44^. As such, we might expect brain regions associated with ToM processes to not only be sensitive to gaze direction, but to increased ratings of enjoyment as well.

## Results

### Behavioural experiment

#### Enjoyment ratings

**Fig. 2:**
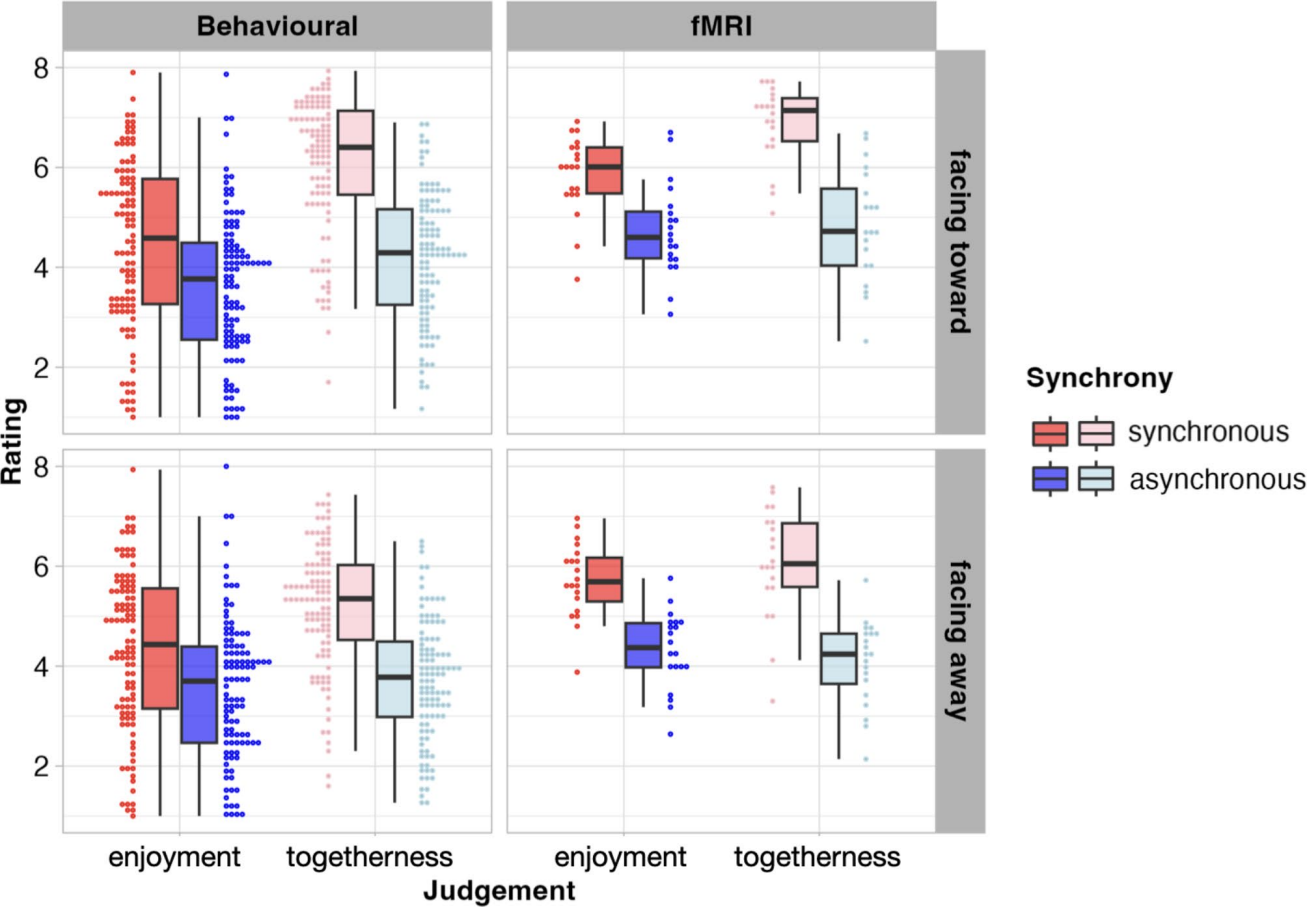
Enjoyment and togetherness ratings for dyads facing toward and away from each other and moving synchronously and asynchronously. Red and pink boxes and dots show behavioural data (2012 and 2023 datasets aggregated; scale from 1–8). Light and dark blue boxes and dots show behavioural responses collected during the fMRI experiment (responses collected on scale from 1–4 and multiplied by 2 to be visualised on same scale as data from the behavioural experiment).

The model predicting enjoyment showed a main effect of synchrony (red bars in left panels of Fig. 2), and a main effect of the year in which data were collected (*p*s < 0.05). The main effect of gaze direction, the interaction between synchrony and gaze direction and remaining predictors (i.e., age and dance experience) were not statistically significant (all *p*s > 0.05). The model explained 7.30% of the variance in the data. Post hoc tests revealed that enjoyment was greater when dyads moved synchronously compared to when dyads moved asynchronously (*p* < 0.001; Fig. 2; further details in Supplementary Tables 1a and 1b).

#### Togetherness ratings

The model predicting togetherness showed a significant effect of the interaction between synchrony and gaze direction, as well as main effects of synchrony and gaze direction, such that synchronous and facing dyads were rated higher on togetherness (pink bars in left panels of Fig. 2), and a main effect of age whereby age positively predicted togetherness ratings (all *p*s < 0.05). We did not find compelling evidence that dance experience was associated with ratings of togetherness (*p* = 0.528). The model explained 36.90% of the variance in the data. Post hoc tests revealed that ratings of togetherness were highest when dyads moved synchronously and faced toward each other compared to all other conditions, and lowest when dyads moved asynchronously and faced away from each other (all *p*s < 0.001, corrected for multiple comparisons; Fig. 2; see details in Supplementary Tables 1a and 1b).

### fMRI experiment

#### Enjoyment ratings

For enjoyment ratings, the model showed a main effect of synchrony and a marginal effect of gaze direction on enjoyment ratings (dark blue bars in right panels of Fig. 2; *p* synchrony < 0.05; *p* gaze direction = 0.059). Neither the interaction between synchrony and gaze direction nor the other predictors (i.e., age and dance experience) reached our predefined threshold of statistical significance (all *p*s > 0.05). The model explained 45.5% of the variance in the data. Post hoc tests revealed greater ratings of enjoyment when dyads moved synchronously compared to when they moved asynchronously (*p* < 0.001). Ratings were numerically, but not significantly, greater when dyads faced toward each other, relative to facing away from each other (*p* = 0.067; see details in Supplementary Tables 2a and b). An exploratory analysis conducted during the review process showed that ratings of enjoyment were positively correlated with ratings of togetherness (*p* < 0.001), when controlling for all other variables.

#### Togetherness ratings

For togetherness, the model revealed main effects of synchrony and gaze direction (light blue bars in right panels of Fig. 2), as well as age and dance experience (all *p*s < 0.05). Here we observed that age predicted togetherness ratings negatively, whereas dance experience positively predicted togetherness ratings. The interaction between gaze direction and synchrony was not statistically significant (*p* > 0.795). The model explained 63.7% of the variance in the data. Post hoc tests revealed that ratings of togetherness were higher when dyads moved synchronously compared to when dyads moved asynchronously (*p* < 0.001). Further, ratings of togetherness were greater when dyads faced toward each other than when they faced away (*p* < 0.001; Fig. 2; see details in Supplementary Tables 2a, b).

#### Imaging results

##### Whole-brain and region-of-interest analyses

**Fig. 3:**
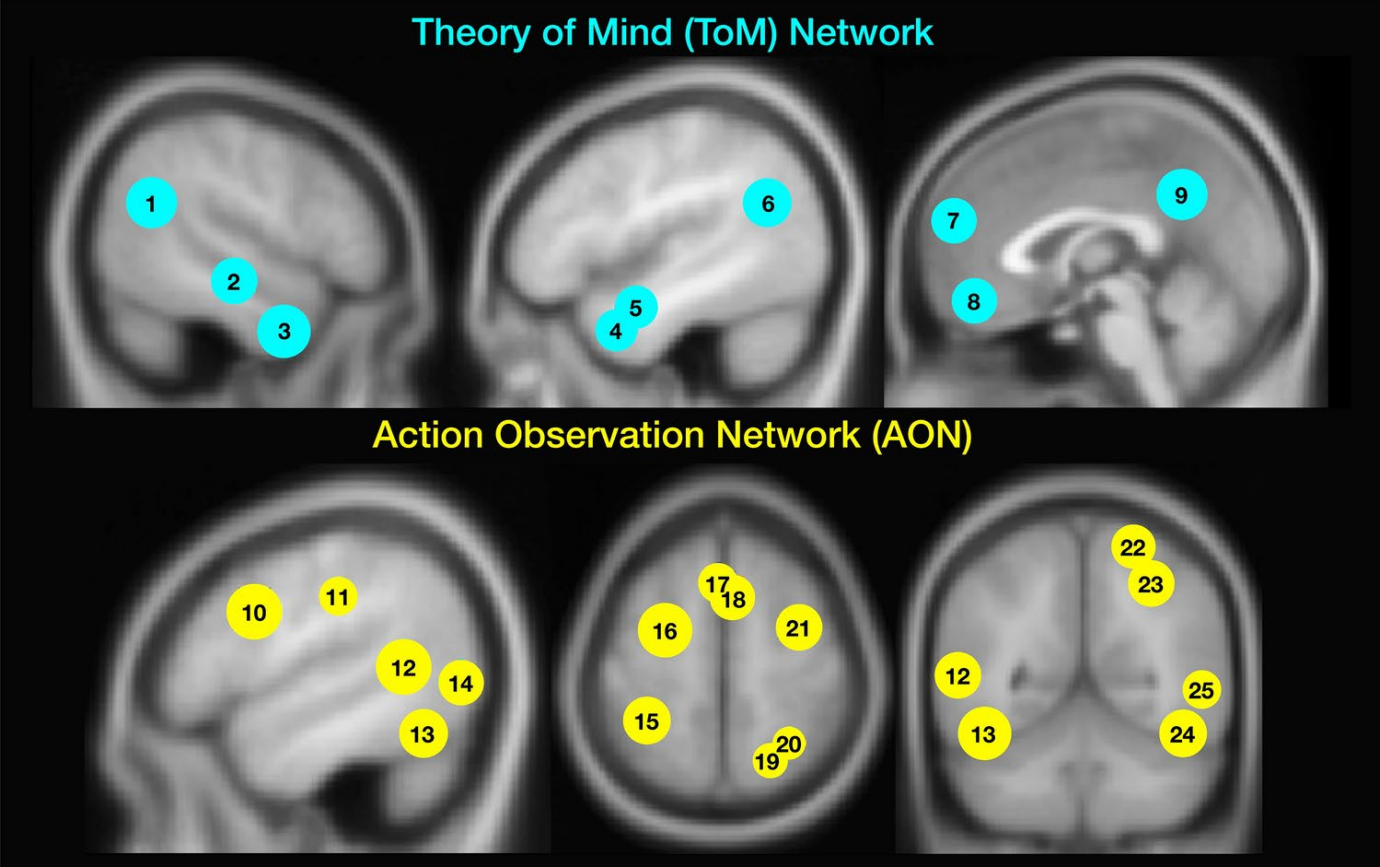
Regions of interest (ROIs) belonging to the Action Observation (AON) and Theory of Mind (ToM) networks. Numbers for each network correspond to the following brain regions: Theory of Mind Network: 1 = Right Temporoparietal Junction (R TPJ); 2 = Right Superior Temporal Sulcus (R STS); 3 = Right Temporal Pole (R TP); 4 = Left Temporal Pole (L TP); 5 = Left Superior Temporal Sulcus (L STS); 6 = Left Temporoparietal Junction (L TPJ); 7 = (Midline) Dorsomedial Prefrontal Cortex (dmPFC); 8 = (Midline) Ventromedial Prefrontal Cortex (vmPFC); 9 = (Midline) Precuneus. Action Observation Network: 10 = Left Inferior Frontal Gyrus (L IFG); 11 = Left Inferior Parietal Lobule (L IPL); 12 = Left Superior Temporal Sulcus (L STS); 13 = Left Fusiform Gyrus (L FG); 14 = Left Lateral Occipital Complex (L LOC); 15 = Left Primary Somatosensory Cortex (L SI); 16 = Left Dorsal Premotor Cortex (L dPMC); 17 = Left Supplementary Motor Area (L SMA); 18 = Right Supplementary Motor Area (R SMA); 19 = Right Superior Parietal Lobule (R SPL); 20 = Right Inferior Parietal Sulcus (R IPS); 21 = Right Dorsal Premotor Cortex (R dPMC); 22 = Right Primary Somatosensory Cortex (R SI); 23 = Right Inferior Parietal Lobule (R IPL); 24 = Right Fusiform Gyrus; 25 = Right Superior Temporal Sulcus (R STS); Right Occipital Complex (R LOC) not visualised. Coordinates for all ROIs are listed in Supplementary Table 3.

Results from the whole-brain and region-of-interest (ROI) analyses showed comparable results. We focus on the ROI analyses in the main text of the paper and report whole-brain analyses in the Supplementary Materials to avoid repetition (Supplementary Table 3). ROIs are visualised in Fig. 3.

##### Action observation network ROIs

**Fig. 4:**
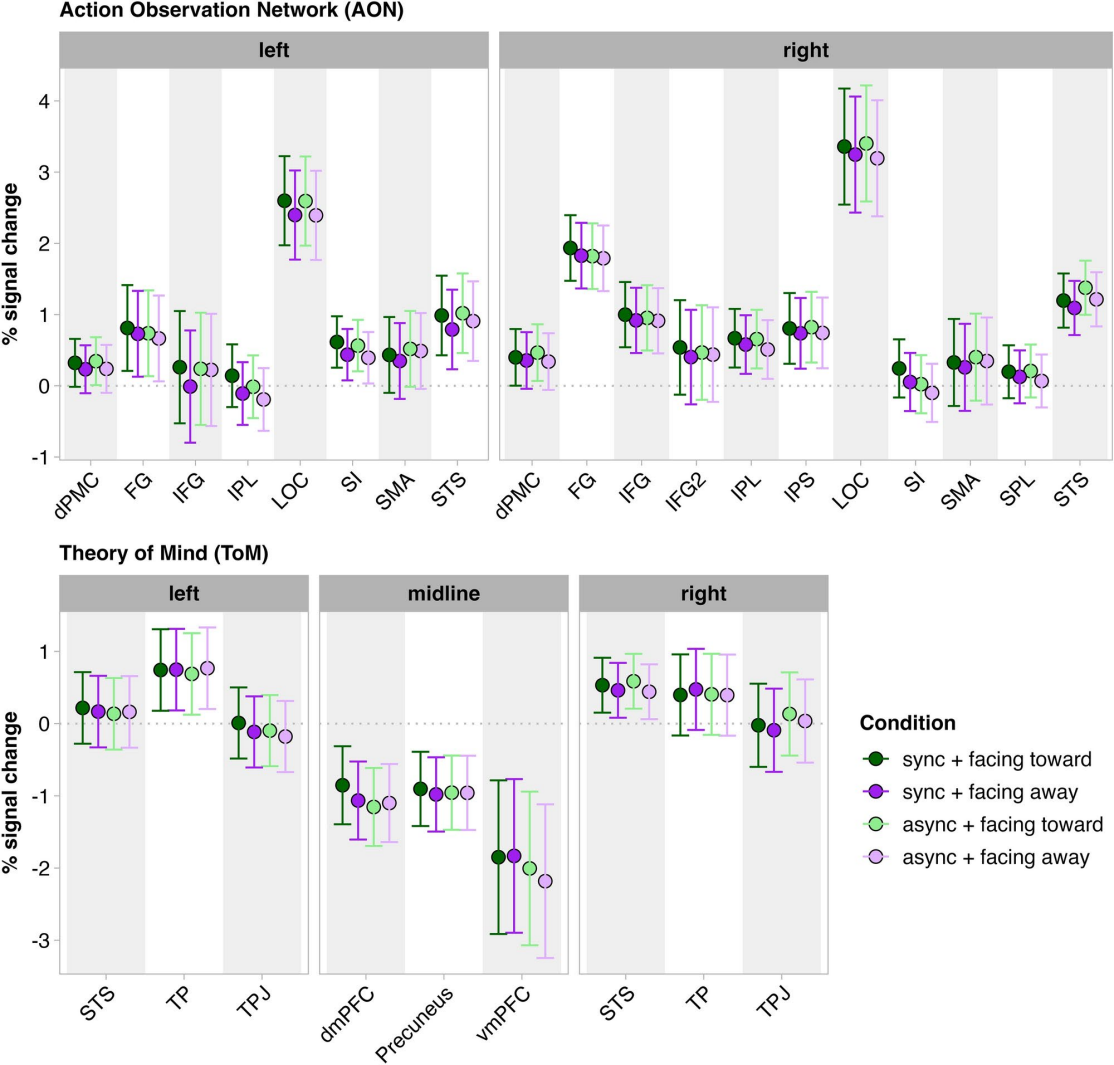
Percentage of signal change in ROIs belonging to the Action Observation Network (AON) and Theory of Mind (ToM) network when dyads are facing toward vs. away from each other (green vs. purple), and when moving in synchrony vs. out of synchrony with each other (deeper vs. lighter hues). Error bars represent 95% confidence intervals.

For each ROI in the AON, we fit a separate model (Supplementary Tables 4a–d). Our analyses revealed a main effect of gaze direction in the left IFG, right SPL, and bilaterally in the IPL, SI, STS, LOC, and dPMC (all *p*s < 0.05) such that responses in these ROIs were higher when dyads faced toward each other compared to when they faced away (Fig. 4). We found a main effect of synchrony in the left IPL (*p* = 0.018) and right SI (*p* < 0.001), whereby responses were greater when dyads moved synchronously compared to when they moved asynchronously. We found the opposite pattern of responses in left SMA (*p* = 0.039) and right STS (*p* = 0.002), where responses were higher for asynchronous, compared to synchronous movements dyads (Fig. 4). Age positively predicted the response in the right STS (*p* = 0.027). The interaction between synchrony and gaze direction was significant only in left IFG (*p* = 0.028). Post hoc tests suggested that the effect of gaze direction (facing toward > facing away) was significant only when dyads were moving synchronously (*p* = 0.001) but not when dyads were moving asynchronously (*p* = 0.85; Fig. 4).

##### Theory of Mind Network ROIs

We also fit separate models per ROI belonging to the ToM network (Supplementary Tables 5a,b). Results showed a main effect of gaze direction in the right STS such that the response was higher when dyads faced toward each other relative to away (*p* = 0.025; Fig. 4). We found a main effect of synchrony, whereby responses in the vmPFC (*p* = 0.048) and dmPFC (*p* = 0.049) were greater when dyads moved synchronously compared to asynchronously. In the right TPJ, the response was greater when dyads moved asynchronously compared to synchronously (*p* = 0.004). No other main effects (i.e., age and dance experience) emerged, nor did the interaction between synchrony and gaze direction reach significance within any of the ToM network ROIs.

#### Link between ROI responses and enjoyment and togetherness ratings

To test whether ROI responses varied as a function of enjoyment ratings, we fit additional models per ROI with the three-way interaction of enjoyment, synchrony, and gaze direction. We then did the same for togetherness. These models allowed us to test whether an increase in enjoyment and/or togetherness ratings predicts an increase (or decrease) in ROI responses as a function of synchrony and/or gaze direction.

**Fig. 5:**
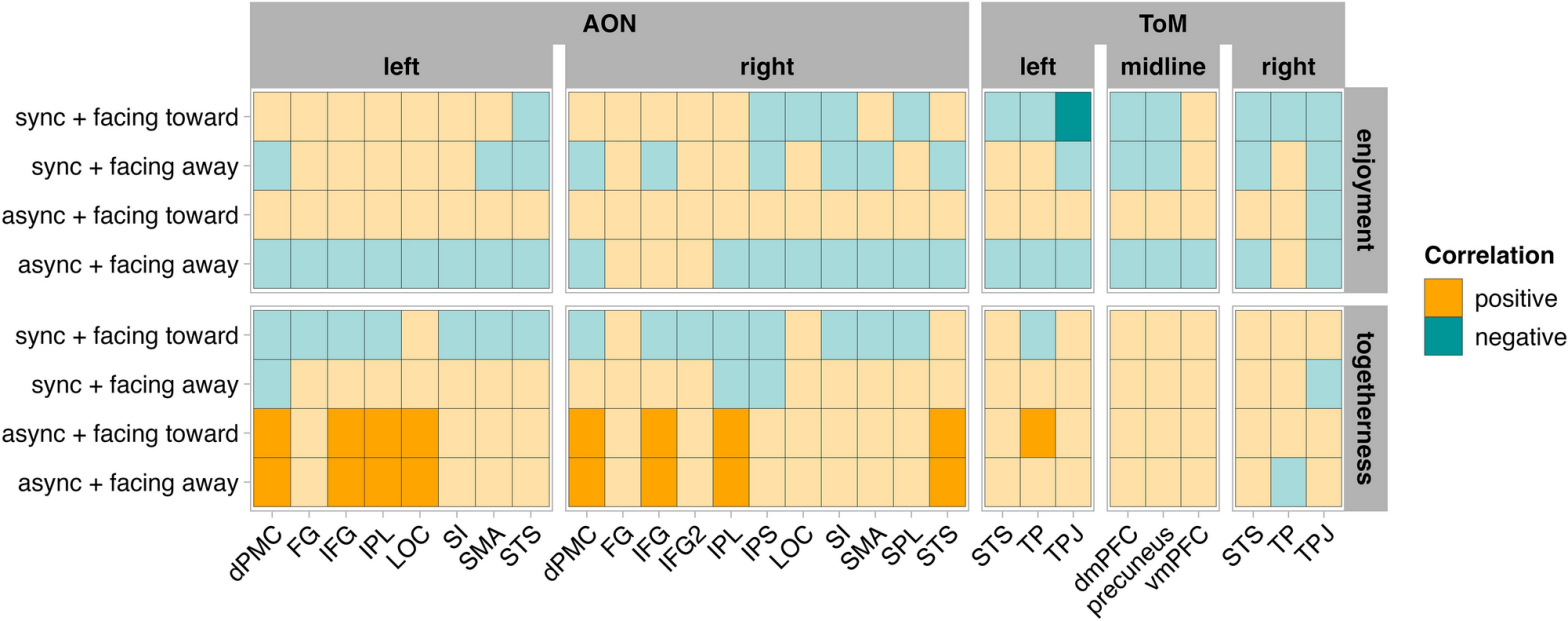
Correlations matrix showing relationships between condition, brain activity in each ROI, and participants’ ratings (i.e., the 3-way interactions). Yellow squares reflect positive correlations and turquoise squares represent negative correlations. The transparency represents significance; more transparent squares indicate *p* values > 0.05 and solidly filled squares indicate *p* values < 0.05.

We found that the interaction between enjoyment, synchrony, and gaze direction did not predict any of the AON responses (all *p* values > 0.05; Supplementary Tables 6a–d). The interaction between togetherness and synchrony predicted responses in bilateral IFG, IPL and dPMC, and left LOC and right STS (all *p* values < 0.05; Supplementary Tables 7a–d). Post hoc analyses suggested that many of the AON ROI responses were positively predicted by togetherness ratings, but only in the asynchronous condition and not the synchronous condition (all *p* values < 0.05; Fig. 5).

For the ToM regions, the three-way interaction between enjoyment, synchrony, and gaze direction predicted responses in the left TPJ (*p* = 0.034; Supplementary Tables 8a, b). Post hoc tests revealed that the response in the left TPJ was marginally weaker the more highly participants rated their enjoyment of the dancers, but only when dancers moved synchronously and faced toward each other (*p* = 0.052; Fig. 5), and not for any other condition (all *p* values > 0.05). The three-way interaction between togetherness, synchrony, and gaze direction predicted responses in the left TP (*p* = 0.047; Supplementary Tables 9a, b). Post hoc tests suggested that response in the left TP was stronger the more highly the dancers were rated as being together, but only when dancers were moving asynchronously and facing toward each other (*p* = 0.049) and not for any other condition (all *p* values > 0.05; Fig. 5).

## Discussion

With this study, we sought to examine the impacts of mutual gaze and movement synchrony on observers’ evaluations of two dancers’ togetherness, as well as observers’ enjoyment of the dancers’ movements. Togetherness ratings reflect the degree of joint or shared agency that the dancers exhibit^15^, i.e., how much the dancers are perceived as being aware of each other and working as a pair. Behaviourally, participants assigned higher ratings of togetherness to video displays that depicted dyads who faced each other or moved synchronously. Moreover, togetherness ratings were highest when dance movements featured movement synchrony and mutual gaze. For ratings of enjoyment of the dance movements, participants assigned higher values to synchronous than asynchronous dance movements, and no effects of gaze direction were detected on overall enjoyment ratings.

Our neuroimaging findings highlight the impacts of gaze direction and synchrony on engagement of ROIs drawn from the AON and ToM networks, revealing widespread sensitivity to gaze direction among bilaterally distributed AON regions, and the right STS from the ToM network. Contrary to our expectations, synchrony did not drive engagement of many of the ROIs drawn from the AON or ToM network. Only the left IPL and right SI from the AON and dorsal and medial portions of PFC from the ToM network demonstrated more robust responses when the dancers moved synchronously. We additionally observed that left SMA and a portion of the right STS from the AON and the right TPJ from the ToM network respond more robustly when the dancers moved asynchronously. Note that the STS ROIs defined for the AON and ToM networks were distinct clusters (see Fig. 3 and Supplementary Table 10 for details). Finally, one region within the AON, the left IFG, showed an interaction between synchrony and gaze direction, such that effects of gaze direction only manifest when the dancers moved in sync with one another. Below we consider the implications of our behavioural and imaging findings in turn, as well as the relationship between ROI responses and enjoyment and togetherness ratings.

### Synchrony predicts ratings of enjoyment and togetherness, while gaze predicts ratings of togetherness

We present behavioural results from three samples (outside the fMRI scanner in 2012 and 2023 [aggregated in our analyses], and inside the fMRI scanner in 2012; see “Methods” section for details). Across the three samples, observers assigned greater ‘togetherness’ to synchronous than asynchronous dance duets. We conceptualise togetherness as the extent to which the dancers are seen to be working as a pair, and how aware the dancers are of each other. Our finding that synchronous dance–or movement–is associated with higher ratings of togetherness is consistent with previous reports on the perceptual consequences of movement synchrony^13–15^. Furthermore, this finding adds support to the notion that at least some of the behavioural consequences for those who engage in movement synchrony (e.g., rapport, social closeness, affiliation^3,8,9,45^) may also be inferred when *watching* dyads engage in movement synchrony.

We also found that synchronous movements were associated with greater aesthetic value (i.e., enjoyment) of dance duets. Our findings pertaining to enjoyment align with earlier work exploring the impact of synchrony on aesthetic perception of movement, using pairs of hip-hop dancers^12^, non-dancers performing upper body movements^10^, and larger ensembles of dancers moving in and out of synchrony^11^. Accordingly, our findings confirm that synchrony shapes aesthetic judgements of human movements. More broadly, it is possible that observers experience greater enjoyment of synchronous movements in contexts where synchrony enhances collaboration (i.e., playing the mirror game^10^, keeping up with a group fitness class, or paddling a dragon boat). It follows that observers may experience less enjoyment of synchronous movements in contexts where high levels of synchrony can hinder successful collaboration (i.e., packing a picnic basket, performing in jazz concerts or improv theatre). Yet, if we focus on the aesthetic experience of observing dance, synchrony is not to be confused with “successful collaboration” (for example, synchrony does not play a strong role in contact improv dance, in fact, departures from synchrony are desirable). In a study by Vicary et al.,^11^ where dancers moved in and out of synchrony in choreographies involving everyday movements such as walking and waving, all degrees of synchrony were intentional, therefore representing successful collaboration. Nonetheless, observers rated choreographies involving less synchrony as less enjoyable. It thus follows that the enjoyment of synchrony is more strongly contingent on the synchrony itself than the perceived ‘success’ of the collaboration.

Ratings of togetherness represent a potentially more nuanced metric with which to probe perceptions of interactions between the dancers. We conceptualise togetherness as the degree to which pairs of dancers form a social unit exhibiting joint agency (i.e., actively collaborating^15^). In our study, gaze direction shaped perceptions of togetherness but did not predict ratings of enjoyment. Observers were more likely to consider dancers part of a social unit with joint agency, i.e., more together, when the dancers’ bodies and faces were oriented toward each other. Given that mutual gaze is a robust cue to social interaction^25–27^, this finding is not surprising. On the other hand, we had reasonable grounds to predict that mutual gaze could enhance enjoyment of dyadic movements^41,43,44^. Yet this effect was not borne out in our behavioural experiment, and the ratings collected during the fMRI experiment showed only a trend towards greater enjoyment when mutual gaze was present. It is plausible that our video-editing choices dampened possible differences in enjoyment based on gaze direction. Specifically, the dancers were filmed facing each other while performing different choreographic sequences, and we then edited the original footage so that the movements occurred asynchronously and/or the dancers’ positions were swapped to make them back-to-back. To make all four conditions of our design as visually similar as possible, we placed a black bar between the two dancers for all conditions (Fig. 1). We acknowledge that the introduction of a black bar between the videos of the two dancers might have interrupted or attenuated the strength of the mutual gaze cues, thereby reducing their impact on ratings of enjoyability or aesthetic value. However, our aim, for experimental control, was that no one condition appeared more normal or veridical. We do acknowledge that videos from the synchronous mutual gaze condition may contain subtle cues (beyond mutual gaze) that could influence our measures, since this was the only condition where the dancers were neither flipped nor time-delayed. It nonetheless remains an open question for future work (and one particularly relevant for choreographers and dancers) to examine how the pattern of results presented here would change if dance sequences (matching our experimental conditions) were filmed in a single frame for all conditions, with the dancers modulating their synchrony and gaze patterns according to choreographic intent.

Finally, we also observed an interaction between synchrony and gaze direction in the behavioural experiment for togetherness ratings, such that togetherness ratings were highest for synchronous movements with mutual gaze. As synchrony and gaze direction index the relationship between people^13,14,25–27^, this finding is in line with our expectations and prior research. It follows that the combination of synchrony and mutual gaze defines, or signals, that the dancers belong to one social unit and/or share a goal. Moreover, this suggests that the superimposed black bar between the dancers did not fully interrupt participants’ (self-reported) perceptions of perceiving the dancers as socially connected duets at the behavioural level. Nonetheless, these findings would benefit from being replicated with other stimuli, wherein two dancers dance in synchrony, facing toward and away from one another with no additional video manipulation involved. Moreover, an additional pair of conditions could be considered, in which dancers both face the same direction, or with one dancer inverted, and moving either synchronously or asynchronously. Such conditions could be useful for isolating the contributions of visual features such as symmetry from synchrony per se.

### AON brain regions more sensitive to differences in gaze than synchrony

Our main research questions focused on activity within ROIs specific to the AON and ToM network. In the following, we focus on the implications of the ROI findings. For the interested reader, a brief discussion of our whole-brain imaging findings is included in the Supplementary Materials.

Brain regions associated with the AON, including left IFG, bilateral IPL, SI, STS, LOC and dPMC, showed a more robust response when the dancers faced each other than when they faced away from each other. We were not surprised to see that these two regions responded more robustly to mutual gaze, given the literature implicating a portion of the posterior STS in indexing social interactions (or social relationships) between dyads^31–33^, as well as a portion of the LOC indexing dyads facing toward each other, relative to back-to-back^34,35^. The more anterior sensorimotor brain regions, however, have not previously been reliably implicated in mutual gaze or social interaction perception per se. Recent EEG research documents how two bodies facing each other, as opposed to pairs of bodies facing away and non-animate objects facing toward or facing away, are preferentially encoded^46^, demonstrating that facing, or interacting, humans are processed by the brain as much more than the sum of their parts (i.e., objects with faces)^46^. Following from this recent work, we propose that the patterns of activation extending beyond occipitotemporal components of the AON could be evoked by the perceived socialness of facing dyads–though this remains to be addressed empirically in future research.

Based on recent functional near infrared spectroscopy (fNIRS) findings from our group^24^, we anticipated more robust engagement of bilateral frontotemporal AON regions (IFG and STG) when participants observed dancers moving in synchrony. However, the design of this prior work did not allow for an asynchronous condition to included. The present study fills this gap, revealing that left IPL and right SI are sensitive to synchronous, as opposed to asynchronous, movements. Returning to Shamay-Tsoory’s extended integrative model of alignment^17^, as well as evidence that observers are likely to simulate the synchronous movements they observe^19–22^, these sensorimotor regions play an important role in upregulating coupling between perceived and performed movements. It thus seems plausible that some degree of sensorimotor processing is also called upon during observation and evaluation of synchrony, even when no concurrent actions are being performed. Establishing the contributions of these regions to synchrony perception per se will require further investigation, ideally involving manipulation of both synchrony (synchronous vs asynchronous movements) and performance (physical execution vs observation).

The final finding to emerge from the AON concerns the interaction between gaze direction and synchrony within the left IFG. Left IFG responded more strongly to gaze direction when dyads moved synchronously than when they moved asynchronously (top panel of Fig. 4). One explanation for this finding is that just as some occipitotemporal portions of the AON have been identified as sensitive to perceiving mutual gaze in dyads^35,36^, this more anterior/motor portion of the AON might knit together cues of socialness across multiple channels (in this case, gaze direction and movement kinematics). Alternatively, IFG may be tuned to mutual gaze and/or movement asynchrony, and thus responds less when neither of these cues are present. This interpretation would align with findings reported by Georgescu and colleagues, who manipulated movement contingency and smoothness between pairs of facing dyads^18^. Georgescu et al. found a portion of right IFG to be most responsive to contingent (non-mirrored, asynchronous) compared to mirrored (i.e., synchronous) movements. This interpretation remains speculative at this stage, and further research will be required to fully characterise the interaction between movement synchrony and gaze direction at the brain level.

### ToM network indexes synchrony within dyad

The overall pattern of findings reported from the ToM network was in line with some of our original hypotheses, and contradicted others. Specifically, we found that right STS responded more robustly to dancers engaged in mutual gaze than dancers facing away from each other. As past work has shown the engagement of the STS to be a strong predictor of social engagement between individuals^31–33,35,36,46^, we were not surprised to see the STS show a more robust response to mutual gaze. However, the previous literature examining STS’s role in social interaction perception has almost exclusively focused on posterior STS^31–33,35,36,46^. Our ToM STS region is in the anterior STS (and indeed, compared to the STS ROI in the AON that we examined in this study, this region is situated in a more inferior, anterior and medial position, and is separated by 27 mm; see regions 5 and 12 in Fig. 3). We also report evidence that the right TPJ was not sensitive to gaze direction, but instead responded more robustly to asynchronous than synchronous movements.

Deen and colleagues offer further insight into the contributions of STS and TPJ, within the ToM network, to social perception^47^. The authors probed responses along the length of the STS using separate theory of mind, language, voice, face, and biological motion perception tasks, to better understand its functional organisation^47^. They found that viewing biological motion engaged a more anterior portion of the STS relative to theory of mind tasks, and that perceiving dynamic faces engaged a more anterior portion of STS than bodies. Mutual eye gaze may have drawn our participants’ attention to the dancers’ faces (which were fully visible), thereby driving the activation of anterior right STS. We also lean on Deen et al.’s^47^ mapping of the STS when suggesting that the right TPJ’s stronger response to asynchronous than synchronous movements could be driven by increased biological motion processing. Our reasoning is the attentional demands of tracking two bodies moving asynchronously may be greater^48^ or may require more demanding inferential computation to understand why the dancers are moving asynchronously^49^. An opportunity exists for future work to further explore these possibilities.

The final result to consider from the ToM network concerns the opposite pattern: greater responses to synchronous than asynchronous movements. Portions of both the dorsal and ventral medial prefrontal cortex (dmPFC and vmPFC; centered on the midline) showed this response profile. We return to Shamay-Tsoory and colleagues’ model for maintaining forms of interpersonal alignment, including movement synchrony^17^. When individuals engage in a high degree of movement synchrony, a collection of brain regions implicated in reward processing (including the ventromedial prefrontal cortex; vmPFC) come online, ostensibly in response to the pleasantness or enjoyment brought about by not having fallen out of sync with the other person. The role for prefrontal cortex (including dorsomedial and ventromedial portions) when engaging in movement synchrony and experiencing the social reward has been further corroborated by recent work involving brain imaging with fNIRS^50–52^. Our data are among the first to show that watching synchronous dyadic movements enhances the engagement of these regions compared to asynchronous movement. However, as we discuss in the following paragraph, our data do not enable us to conclude that enjoyment ratings (which can be thought of as one measure of the reward value observers derive from these movements) predict responses within the dorsal and ventral portions of the mPFC (see also Fig. 5). As such, it is possible these portions of the mPFC are performing another role than encoding the reward value for observers, or the relationship between movement synchrony, self-reported enjoyment, and mPFC engagement is more subtle, nuanced, or complex than what we can capture with the present study.

### Ratings of togetherness predict activity in AON, but only for asynchronous movements

The final set of results to consider concerns the correlations between the behavioural evaluations of movement enjoyment and togetherness, and the response profile within our AON and ToM regions of interest (Fig. 5). While we cannot endorse reading too deeply into any of the patterns reported within this correlation matrix given our limited sample size, we might nonetheless speculate on possible mechanisms underpinning these relationships, as a starting point for future research. Ratings of togetherness were associated with brain activity in several AON regions (bilateral dmPFC, IFG, IPL; left LOC and right STS), and only one ToM region (left temporal pole). Within the AON regions, we saw this pattern of activation to be specific to asynchronous movements, regardless of gaze direction. Given the reasonable overlap between this list of AON regions and the AON regions reported by Georgescu and colleagues^18^ as more engaged when participants watched contingent (asynchronous) movements, we cautiously suggest that any time participants intuited or perceived some measure of togetherness (i.e., joint or shared agency) among dancers moving asynchronously, these brain regions were reliably contributing to encoding or driving this togetherness perception.

Correlations between togetherness ratings and ToM engagement revealed that the response within the left temporal pole ROI correlated with togetherness ratings, but only when the dancers moved asynchronously and faced each other. Given the variety of high-level cognitive functions that have been ascribed to the temporal poles, including social cognition, sensory perception, semantic processing, and emotion^53,54^, it is plausible that this region is performing a similar calculation about social chunking that the AON ROIs discussed above are, but unlike the AON regions, the temporal pole only contributes to this processing when mutual gaze is involved.

Relative to ratings of togetherness, ratings of enjoyment correlated with activation in fewer brain regions. In fact, only a left TPJ region identified as part of our theory of mind network demonstrated a negative relationship between reported enjoyment and activation, and only for synchronised movements where the dancers shared mutual gaze. This association between brain activation and aesthetic evaluation is spatially similar to findings from a recent fNIRS study by Moffat and Cross^24^, broadly speaking, who found movement enjoyment to be associated with activation in left STG. However, the direction of the relationship reported by Moffat and Cross was positive, rather than negative. While drawing firm conclusions from a handful of studies would be ill advised even if the results were broadly in agreement, here we can only suggest that the relationship between movement enjoyment/movement aesthetics, movement synchrony and gaze direction remains unclear, and is likely contingent on several factors that differed between the two studies we mention here. A possibly more noteworthy similarity between the present study and that reported by Moffat and Cross^24^ is that measures related to perceiving movement kinematics (synchrony, recognition of movement sequences) are associated with activity across more brain regions than aesthetic ratings.

### General limitations and future directions

One general limitation to note is the possibility that factors extraneous to those we manipulated here (movement synchrony and gaze direction) were more influential in shaping the aesthetic ratings of observers. A rich literature from dance aesthetics documents features of dance choreography that are associated with positive aesthetic evaluations. For example, Cross and colleagues showed a reliable relationship between poorer perceived performance ability and higher aesthetic ratings of a solo dancer’s movements among dance naïve observers^38^, while Orgs and colleagues revealed that a solo dancer’s movements were assigned highest aesthetic evaluations when those movements featured greater spatial symmetry both within the dancer’s body and across a movement sequence, as well as fewer path reversals^55^. Follow-up work documents the importance to aesthetic evaluation of the dancer’s ability to externally rotate their limbs (turn out), and perform movements that demonstrate diagonal spread of the limbs, which give the impression of elongation of the body while challenging the laws of physics^56^. Other recent work evaluated how manipulating the dynamics of a solo dancer’s movements shaped perceivers’ aesthetic evaluations, and showed that faster, more predictable movements with varied velocity profiles were judged to be both less reproducible and more aesthetically pleasing than slower dance sequences with a uniform velocity profile^57^. As the current study was not designed to directly evaluate the impact of the kinematic features and choreographic choices (beyond gaze direction and movement synchrony), we cannot speak to how these other factors may have shaped our results. However, this literature reviewed here highlights that intriguing questions remain to be explored concerning how a wide range of kinematic features might shape and further influence the aesthetic experience of watching dance duets, as most of the work done to date has focused on observation of solo dancers.

We address further methodological limitations pertaining to the measurement of dance experiences, as well as statistical modelling of data at the end of the “Methods” section (section “Limitations of behavioural analyses”).

## Conclusions

From stage to (mobile phone) screen, from folk festivals to nightclubs, dance duets have social and aesthetic repercussions for observers as well as performers. We offer insight into the observers’ perspective, behaviourally and neurally, revealing that movement synchrony influences enjoyment and togetherness evaluations, while mutual gaze has a more marked influence on perceptions of a dyad being a social unit. These findings enhance our understanding of the importance and value of these two major channels of nonverbal social communication, and in some ways point to the primacy of dyadic movement kinematics over gaze direction in social (and aesthetic) signalling at both brain and behavioural levels. Furthermore, this work opens a range of possibilities for future research and experimentation into the social cognitive and aesthetic consequences of these cues for both scientists and performing artists alike.

## Methods

### Open science statement

Across all experiments, we report how the sample size was determined, all data exclusions, and all measures in the study^58^. Following open science initiatives, all raw data and scripts used for analyses are available online for other researchers to pursue alternative questions of interest (https://osf.io/byje6/). For all behavioural data, data pre-processing, statistical analyses, and data visualisations were performed using R^59^ (v4.2.2) and R Studio^60^ (v2022.07.2, Build 576), unless otherwise specified. Linear mixed effects model analyses were executed using the lme4^61^ package (v1.1–31), and post hoc tests were executed using the emmeans^62^ package (v1.8.2). We used an alpha of 0.05 to make inferences and controlled for multiple comparisons using Tukey-HSD in post hoc tests.

### Sample size justification

We collected two behavioural datasets (the first in 2012 and the second in 2023). During the first, data were collected from as many participants as possible over a two-week period (n = 48). For the second, we aimed to double the number of participants collected in similar study^12^, to ensure that the sample would be adequately powered with which to examine the interaction between synchrony and gaze direction (n = 57). As both behavioural studies used the same experimental design (with a few minor changes as detailed below), we aggregated the behavioural datasets for a larger sample size (n = 106), thereby achieving a sample size almost 3 times the size of that previously reported^12^. Separate analyses of the two datasets reveal no major differences in results or demographics (see Supplementary Materials). Nonetheless, we explicitly account for potential differences between these two samples by including year as a categorical variable in our models predicting behavioural outcomes (described in more detail in Sect. 5.3.4).

For the fMRI experiment, conducted in 2012, we recorded as many usable datasets as possible over a three-week period (n = 19).

### Behavioural experiment

#### Participants

In 2012, a total of 50 participants took part in this experiment for monetary compensation (£6) or course credit. The experiment was conducted in-person. All participants provided informed consent and had normal or corrected-to-normal vision. Approval was obtained from the Research Ethics and Governance Committee of the School of Psychology at Bangor University. One participant was excluded because they did not complete the task, and another was excluded because they did not enter their age.

In 2023, 80 participants were recruited online, of which 57 participants completed the task and passed our attention checks. All 2023 participants were offered an opportunity to enter a raffle for one of the ten £20 Amazon vouchers as a reward for their participation. The 2023 behavioural study was approved by the ethical committee at Goldsmiths University of London. Thus, the final behavioural sample included 105 participants (80 women, 24 men, 1 non-binary, M_age_ = 25.10, SD_age_ = 7.49, range = 18–55 years).

All research was conducted in accordance with the Declaration of Helsinki (except for pre-registration). Demographic information for all participants is presented in Supplementary Table 11.

#### Design and Stimuli

A 2 × 2 factorial design was employed to investigate how movement synchrony and gaze direction impact perception (enjoyment and togetherness) of a dyad moving together. This design led to four conditions (Fig. 1):

1. Synchronous and facing toward each other
2. Synchronous and facing away from each other
3. Asynchronous and facing toward each other
4. Asynchronous and facing away from each other

For each condition, 15 stimuli were created. Fifteen dance sequences (7-s long each) were performed in synchrony by two expert dancers facing each other. The dance movement style was a mix between contemporary and street dance, and the movement excerpts were taken from an original piece of choreography developed for a contemporary dance performance. The original 15 stimuli were edited to create 15 new stimuli where the dancers faced away from each other but still moved synchronously. Using the original 15 stimuli, a further 30 new stimuli (15 facing each other, 15 facing away) were created where one of the dancers’ movements lagged by 2 s, so that the pair moved asynchronously. The stimuli are available online (https://osf.io/byje6/).

The experiment was split into three blocks allowing for two rest periods between blocks. Following on a pilot experiment conducted in 2012 to refine the behavioural questions, we asked participants the following questions:

1. How much did you like watching the video?
2. Would you enjoy watching it again in the future?
3. To what extent are the dancers working in a pair?
4. To what extent do you think the dancers are aware of each other?

The first two questions assess enjoyment of the movements and were selected based on prior research in dance aesthetics demonstrating that enjoyment is the aesthetic dimension that indexes aesthetic perceptions of human movement most meaningfully^38,39,57,63^. The latter two questions were formulated to capture the degree to which synchronous movements caused participants to perceive the dyad as a social unit with joint agency^15^.

#### Procedure

Participants who were recruited to complete the behavioural experiment in 2012 were presented stimuli on a Dell PC laptop. Following a fixation cross, each video was played twice in a randomised order, followed by two questions presented sequentially such that participants answered all four questions for each video. Participants had four seconds to respond to each question and responded on an eight-point Likert scale (1 = not at all; 8 = very much) using the laptop keyboard. Following this, all participants completed the Autism Spectrum Quotient questionnaire (ASQ)^64^, Edinburgh Handedness Inventory^65^ and a dance experience questionnaire (see Supplementary Materials). For participants recruited in 2023, three changes were made in the procedure—(1) the stimuli were uploaded on YouTube and presented online via Qualtrics, (2) participants also answered a self-construal questionnaire^66^ at the end of the survey (unrelated to the aims of the current work), and (3) we include catch trials at randomised times to ensure participants were paying attention to the stimuli (e.g., does J come before Z in the alphabet?). The entire experiment lasted no longer than sixty minutes for all participants.

#### Data analysis

Behavioural data were analysed using a linear mixed effects model approach. Our dependent variables were ratings of enjoyment and togetherness. Ratings of enjoyment were calculated as an average of ratings on questions 1 and 2 (‘How much did you like watching the video?’ and ‘Would you enjoy watching it again in the future?’). Ratings of togetherness were calculated as an average of ratings on questions 3 and 4 (‘To what extent are the dancers working in a pair?’ and ‘To what extent do you think the dancers are aware of each other?’).

To investigate whether synchrony and gaze direction influence ratings of enjoyment or togetherness, we constructed models (separately for enjoyment and togetherness) with the interaction between gaze and synchrony as a fixed effect in the model with subject IDs as a random effect: *enjoyment|togetherness* ~ *1* + gaze direction*synchrony + *dance experience* + *age* + *year* + *1|sID*. We opted to model the Likert-scale responses as pseudo-continuous based on examples from Robitzsch^67^, as well as Sullivan and Artino^68^. We included dance experience and age as continuous variables and the year in which data was collected (2012 or 2023; categorical variable) as fixed effects to test whether any interaction between gaze and synchrony would persist above and beyond the control variables of participant age, dance experience, and date of data collection. For measuring dance experience, we asked participants for their frequency of watching a dance performance, social dancing, self-rating of their ability as a dancer, and details about the dance styles they learned (see the Supplementary Materials for more details on the questions asked, and how a composite score for dance experience was calculated).

Age and dance experience were centred to the mean by subtracting the mean from every value of the variable. Both gaze direction and synchrony were added as categorical variables, with ‘synchronous’ and ‘facing toward’ coded as 0.5, and ‘asynchronous’ and ‘facing away’ coded as – 0.5.

### fMRI experiment

#### Participants

Twenty-two participants were recruited from the Bangor community and were either reimbursed with £15 or three course credits for their participation. Informed consent was obtained in line with the guidelines set by the Research Ethics and Governance Committee of the School of Psychology at Bangor University. All participants were right-handed, were not on any medication, did not report neurological damage, and had normal or corrected-to-normal vision. Data from two participants were excluded due to technical problems, and an additional participant did not complete the task. The final sample included 19 participants (9 women, 10 men, M_age_ = 22.74, SD_age_ = 2.50, range = 20–29 years). All research was conducted in accordance with the Declaration of Helsinki, in the exception of pre-registration. See demographic information for all participants in Supplementary Table 11.

#### Stimuli, design, and procedure

Stimuli, design, and procedures were similar to the behavioural experiment, except those participants who completed the rating task inside the fMRI scanner used a 4-point scale (as opposed to the 8-point scale used in the behavioural measures). The entire experiment lasted approximately 1.5 h. Participants completed two functional runs of the rating task (each run lasting approximately 7 min), followed by an anatomical scan and a diffusion tensor imaging (DTI) scan, which was unrelated to the current study.

Results from the behavioural study indicated that the two questions assessing ‘liking’ or enjoyment, and the two questions assessing ‘togetherness’ showed a similar pattern of results. To reduce the number of questions answered while participants were in the fMRI scanner, participants were randomly assigned to answer two questions (one for enjoyment and one for togetherness). Half of the participants responded to ‘How much did you like watching the video?’ and ‘To what extent are the dancers working in a pair?’, and the other half responded to ‘Would you enjoy watching it again in the future?’ and ‘To what extent do you think the dancers are aware of each other?’. Videos were presented in random order and followed by one question. To collect responses to both questions (enjoyment and togetherness), each unique video was presented twice per run. Participants again had 4 s to respond using four-point scale which mapped onto the scanner-compatible button box with four buttons. Whether 1 to 4 indicated high to low or low to high was counterbalanced between participants. Participants were also asked three test questions randomly during the experiment to ensure they were paying attention: “Did at least one dancer turn around?” OR “Did at least one dancer bring her arms over her head?” OR “Did at least one dancer jump?” After the scanning session, participants completed the Edinburgh Handedness Questionnaire, the ASQ, and the dance experience questionnaire.

#### fMRI data analysis

##### Behavioural data

Behavioural data from the scanner was analysed in the same way as the data collected for the behavioural experiment, except that ‘year’ was excluded, as all fMRI data were collected in 2012.

##### fMRI data acquisition

Participants were placed supine in a 3-T Philips MRI scanner using a SENSE 32-channel phased array coil. They were requested to avoid head motion during the scanning session and watched stimuli on a computer screen placed behind the scanner made visible by a mirror attached to the head coil. Responses on the task were recorded with the help of a button box with four buttons each of which represented 1, 2, 3, and 4 on the Likert scale. Thirty axial slices were acquired in an ascending order using a T2*-weighted EPI sequence. The reference slice for slice time correction was the slice acquired in the middle of the sequence (Slice 15). Parameters are as follows: voxel size = 3 × 3 × 3 mm, repetition time = 2000 ms, echo time = 30 ms, flip angle = 80°, field of view = 192 × 192 mm^3^. 409–450 volumes per participant were collected on the task.

Four dummy scans collected at the beginning of each run of the task were not included in any analyses. A high-resolution T1-weighted anatomical image was also collected with the following parameters: repetition time = 2500 ms, echo time = 33 ms, flip angle = 80°, voxel size = 2 × 1 × 1 mm^3^.

##### Data pre-processing and general linear model

Functional images were preprocessed in SPM-8. Data were realigned, unwarped, and corrected for slice timing. Data were normalised to the Montreal Neurological Institute (MNI) template with a resolution of 3 mm^3^, and images were spatially smoothed (8 mm) and high pass filtered (128 s).

For the main task, a design matrix was fit for each participant with eight regressors: One each for the four main conditions of the task, and one each for the fixation, response, test video and test response.

##### Whole-brain analyses

Contrast images (synchronous > asynchronous; facing toward > facing away) were calculated at the single-subject level. Group-level contrast images were created from these single-subject contrast images to identify regions that were consistently engaged for synchrony and gaze direction across the sample using one-sample t-tests. To identify a neural signature of the interaction of synchrony and gaze direction on action perception, a Synchrony × Gaze direction ANOVA was computed (synchronous [facing toward > facing away] > asynchronous [facing toward > facing away]) as we expected that stimuli where the dancers move synchronously and face each other would show higher activation in the AON and ToM networks. For all analyses, contrast images were taken to the group level and thresholded using a voxel-level threshold of p < 0.001 and a voxel extent of 10 voxels. Correction for multiple comparisons was performed at the cluster level^69^, with clusters that survive correction for multiple corrections using a family-wise error correction. This restricts the likelihood of false positives^70^. Clusters of activity were identified with the SPM Anatomy toolbox^71–73^. Results for these analyses are presented in Supplementary Table 3.

##### Region of interest (ROI) analyses

Region-of-Interest (ROI) analyses were performed using the MarsBar Toolbox in SPM8^72^. Parameter estimates were derived for each of our four conditions (Fig. 1) compared to baseline.

We complemented the whole brain approach with ROI analyses as ROI analyses provide more power over whole-brain approaches to detect small effects in task-related brain regions. To extract parameter estimates, two sets of ROIs were defined: AON network ROIs that are associated with the observation of action^74^, as well as ToM network ROIs that are associated with mentalizing^75^. For the AON, we created spheres (10 mm) using MNI coordinates from a pre-existing meta-analysis on the AON for regions that were engaged for action observation consistently across numerous studies^74^. A total of 19 ROIs were defined as being part of the AON. These ROIs included left IFG, two regions in right IFG, bilateral IPL, right SPL, bilateral SI, right IPS, two regions in the SMA, bilateral lateral occipital gyrus, bilateral fusiform gyrus, bilateral lateral dorsal premotor cortex, and bilateral STS (Fig. 3). For the ToM network, we created spheres (10 mm) using MNI coordinates from work by Jacoby and colleagues^76^. We used MNI coordinates reported for the False Belief > False Photo contrast and identified a total of 9 ROIs: bilateral STS, bilateral TPJ, dMPFC, vMPFC, bilateral TP, and precuneus (Fig. 3). MNI coordinates for all ROIs in Supplementary Table S3. Parameter estimates were extracted from each ROI separately for both the AO and ToM networks.

We first used the same linear mixed effects model for each ROI, as for the behavioural data: *ROI response* ~ *1* + gaze direction*synchrony + *age* + *dance experience* + *1|sID*. To examine the link between brain activity and behaviour, we subsequently fit the same models with an added 3-way interaction between synchrony, gaze direction and participants’ ratings (either enjoyment or togetherness).

### Limitations of behavioural analyses

We further acknowledge that individual differences such as age and dance experience might all have meaningful influences on aesthetic preferences. As the current study is not sufficiently powered to explore such individual differences, we would encourage future work to do so with larger sample sizes and a (pre-registered) design specifically aimed at investigating these variables.

A further limitation to note concerns our approach for assessing dance experience. We found that participants’ dance experience predicted ratings of togetherness, in that greater reported dance experience was associated with higher ratings of togetherness. However, we cannot easily situate our participants within the broader population in terms of how dance savvy or naïve they are, as we used a simple bespoke survey that has not been validated with the population at large. Since the original study was run, the Goldsmiths Dance Sophistication Index (Gold-DSI) has been published^77^, which provides a validated and rigorous research tool to assess and situate participants’ dance sophistication. For researchers interested in the impacts of dance training and engagement on behavioural and neural measures, we encourage the use of the Gold-DSI so that the dance sophistication of individual study samples can be better understood within broader population contexts.

## Supplementary Information

Supplementary Information.

## Data Availability

The datasets collected and analyzed for this study can be found our repository entitled “Double Dance” on OSF: https://osf.io/byje6/.
