# Supplementary material for: Mutual gaze and movement synchrony boost observers’ enjoyment and perception of togetherness when watching dance duets
Source: Sci Rep. 2024 Oct 14;14:24004. doi: 10.1038/s41598-024-72659-7 (PMC11473960; doi:10.1038/s41598-024-72659-7)
Supplement: Supplementary file 1 — Supplementary Information. [file 41598_2024_72659_MOESM1_ESM.pdf]

SUPPLEMENTARY MATERIAL
Delightful Duets: Motor synchrony and mutual gaze enhance dance enjoyment and perceptions of socialness

Supplementary Table 1a. Behavioural experiment – Statistical estimates from the linear mixed effects model, separately for liking and togetherness ratings.

	liking				togetherness			
Predictors	*Estimates*	*CI*	*Statistic*	*p*	*Estimates*	*CI*	*Statistic*	*p*
(Intercept)	4.03	3.08, 4.99	8.29	**<0.001**	4.79	4.50, 5.09	32.23	**<0.001**
sync c	0.82	0.70, 0.93	13.84	**<0.001**	1.68	1.53, 1.83	22.06	**<0.001**
gaze c	0.09	-0.02, 0.21	1.59	0.113	0.69	0.54, 0.84	9.00	**<0.001**
age c	0.10	-0.20, 0.40	0.67	0.505	-0.26	-0.48, -0.04	-2.34	**0.020**
dance c	0.12	-0.14, 0.37	0.91	0.361	-0.06	-0.26, 0.13	-0.63	0.528
sync c * gaze c	0.04	-0.20, 0.27	0.30	0.766	0.37	0.07, 0.67	2.44	**0.015**
**Random Effects**								
σ^2	0.37				0.61			
τ_{00}	1.64 $_{sid}$				0.91 $_{sid}$			
	0.44 $_{year}$				0.02 $_{year}$			
ICC	0.85				0.60			
N	105 $_{sid}$				105 $_{sid}$			
	2 $_{year}$				2 $_{year}$			
Observations	420				420			
Marginal R^2 / Conditional R^2	0.073 / 0.861				0.369 / 0.751			

Supplementary Table 1b. Behavioural experiment – Posthoc tests for liking and togetherness linear mixed models.

Contrast (liking)	estimate	SE	df	t.ratio	p.value
(gaze.c-0.5 sync.c-0.5) - (gaze.c0.5 sync.c-0.5)	-0.0763	0.0839915	318.0272	-0.9090107	0.8000602
(gaze.c-0.5 sync.c-0.5) - (gaze.c-0.5 sync.c0.5)	-0.8004	0.0839915	318.0272	-9.5294935	0.0000000
(gaze.c-0.5 sync.c-0.5) - gaze.c0.5 sync.c0.5	-0.9120	0.0839915	318.0272	-10.8580476	0.0000000
(gaze.c0.5 sync.c-0.5) - (gaze.c-0.5 sync.c0.5)	-0.7240	0.0839915	318.0272	-8.6204829	0.0000000
(gaze.c0.5 sync.c-0.5) - gaze.c0.5 sync.c0.5	-0.8356	0.0839915	318.0272	-9.9490369	0.0000000
(gaze.c-0.5 sync.c0.5) - gaze.c0.5 sync.c0.5	-0.1116	0.0839915	318.0272	-1.3285541	0.5454079

Contrast (togetherness)	estimate	SE	df	t.ratio	p.value
(gaze.c-0.5 sync.c-0.5) - (gaze.c0.5 sync.c-0.5)	-0.3745	0.1097206	63.1579	-3.413214	0.0060448
(gaze.c-0.5 sync.c-0.5) - (gaze.c-0.5 sync.c0.5)	-0.9905	0.1097206	63.1579	-9.027473	0.0000000
(gaze.c-0.5 sync.c-0.5) - gaze.c0.5 sync.c0.5	-1.4045	0.1097206	63.1579	-12.800692	0.0000000
(gaze.c0.5 sync.c-0.5) - (gaze.c-0.5 sync.c0.5)	-0.6160	0.1097206	63.1579	-5.614259	0.0000028
(gaze.c0.5 sync.c-0.5) - gaze.c0.5 sync.c0.5	-1.0300	0.1097206	63.1579	-9.387478	0.0000000
(gaze.c-0.5 sync.c0.5) - gaze.c0.5 sync.c0.5	-0.4140	0.1097206	63.1579	-3.773219	0.0019842

Supplementary Table 2a. fMRI experiment – Statistical estimates from the linear mixed effects model, separately for liking and togetherness ratings.

	liking				togetherness			
Predictors	*Estimates*	*CI*	*Statistic*	*p*	*Estimates*	*CI*	*Statistic*	*p*
(Intercept)	2.58	2.46 – 2.69	43.70	**<0.001**	2.73	2.59 – 2.88	37.83	**<0.001**
Synchrony	0.63	0.49 – 0.77	8.97	**<0.001**	1.01	0.86 – 1.16	13.36	**<0.001**
Gaze Direction	0.13	-0.01 – 0.27	1.91	0.059	0.39	0.24 – 0.54	5.21	**<0.001**
Age	-0.08	-0.20 – 0.04	-1.40	0.165	0.10	-0.05 – 0.25	1.37	0.174
Dance	0.09	-0.03 – 0.21	1.56	0.124	-0.17	-0.32 – -0.03	-2.35	**0.022**
Synchrony x Gaze Direction	-0.14	-0.42 – 0.14	-1.02	0.310	0.04	-0.26 – 0.34	0.26	0.795
**Random Effects**								
σ^2	0.10				0.11			
τ_{00}	0.05 $_{sid}$				0.08 $_{sid}$			
ICC	0.32				0.40			
N	20 $_{sid}$				20 $_{sid}$			
Observations	80				80			
Marginal R^2 / Conditional R^2	0.455 / 0.626				0.637 / 0.781			

Supplementary Table 2b. fMRI experiment – Posthoc tests for liking and togetherness linear mixed models.

**Contrast (liking)**	**estimate**	**SE**	**df**	**t.ratio**	**p.value**
(gaze.c-0.5 sync.c-0.5) - (gaze.c0.5 sync.c-0.5)	-0.2055	0.1015426	63.15789	-2.0237816	0.1902302
(gaze.c-0.5 sync.c-0.5) - (gaze.c-0.5 sync.c0.5)	-0.6990	0.1015426	63.15789	-6.8838120	0.0000000
(gaze.c-0.5 sync.c-0.5) - gaze.c0.5 sync.c0.5	-0.7615	0.1015426	63.15789	-7.4993173	0.0000000
(gaze.c0.5 sync.c-0.5) - (gaze.c-0.5 sync.c0.5)	-0.4935	0.1015426	63.15789	-4.8600303	0.0000472
(gaze.c0.5 sync.c-0.5) - gaze.c0.5 sync.c0.5	-0.5560	0.1015426	63.15789	-5.4755357	0.0000047
(gaze.c-0.5 sync.c0.5) - gaze.c0.5 sync.c0.5	-0.0625	0.1015426	63.15789	-0.6155054	0.9267725

**Contrast (togetherness)**	**estimate**	**SE**	**df**	**t.ratio**	**p.value**
(gaze.c-0.5 sync.c-0.5) - (gaze.c0.5 sync.c-0.5)	-0.3745	0.1097206	63.1579	-3.413214	0.0060448
(gaze.c-0.5 sync.c-0.5) - (gaze.c-0.5 sync.c0.5)	-0.9905	0.1097206	63.1579	-9.027473	0.0000000
(gaze.c-0.5 sync.c-0.5) - gaze.c0.5 sync.c0.5	-1.4045	0.1097206	63.1579	-12.800692	0.0000000
(gaze.c0.5 sync.c-0.5) - (gaze.c-0.5 sync.c0.5)	-0.6160	0.1097206	63.1579	-5.614259	0.0000028
(gaze.c0.5 sync.c-0.5) - gaze.c0.5 sync.c0.5	-1.0300	0.1097206	63.1579	-9.387478	0.0000000
(gaze.c-0.5 sync.c0.5) - gaze.c0.5 sync.c0.5	-0.4140	0.1097206	63.1579	-3.773219	0.0019842

Whole-brain analyses (associated with Supplementary Table 3)

All results are thresholded at p<.05, FEW-corrected for multiple comparisons, but we report regions engaged at p<.001, uncorrected in the tables and figures for clarity. In a whole-brain analysis, we found a main effect of synchrony (synchronous>asynchronous) in the right occipital and parahippocampal gyri as well as the right IPL. The inverse contrast (asynchronous > synchronous) revealed clusters in the right temporoparietal junction, right middle frontal gyrus, and right precuneus. A main effect of gaze direction (facing toward>facing away) was found in a bilateral network within frontal, premotor, parietal and temporo-occipital cortices. No clusters emerged for the inverse (facing away > facing toward), for the interaction (synchronous (facing toward>facing away) > asynchronous (facing toward > facing away)) or for its inverse.

We further ran all our analyses again using a parametric analysis including the ratings of liking and togetherness in the model. Results were similar to those reported above. Engagement of the left angular gyrus increased with increase in ratings of liking, and engagement of the left superior temporal gyrus increased with increase in ratings of togetherness

Supplementary Table 3. Whole-brain fMRI analyses. Main effects of Synchrony and Eye Contact. Thresholded at p<.001 uncorrected, rows in bold indicate p<.05 FWE corrected. Bolded regions showed significant activation.

				**MNI coordinates**		
**Region**	**Cluster Size**	**P FWE Corr**	**t-value**	**x**	**y**	**z**
**(A) SYNCHRONY (Synchronous > Asynchronous)**						
**Right calcarine gyrus**	**87**	**.036**	**5.13**	**18**	**-94**	**1**
			4.84	24	-85	19
			4.11	36	-85	13
Right parahippocampal gyrus	10	.785	5.79	36	-19	-26
			4.95	30	-13	-20
Right supramarginal gyrus/right IPL	14	.684	4.32	54	-22	37
(B) **GAZE (Facing Toward > Facing Away)**						
**Right supramarginal gyrus/left IPL**	**690**	**<.001**	**10.16**	**-48**	**-31**	**34**
			7.36	-27	-43	52
			7.12	-39	-37	49
**Right SPL**	**489**	**<.001**	**7.14**	**21**	**-52**	**58**
			6.84	57	-19	34
			5.95	30	-43	55
**Left SFG**	**103**	**.004**	**7.02**	**-24**	**-10**	**52**
			4.80	3	-10	49
			4.28	-15	-7	55
**Left MTG**	**304**	**<.001**	**6.77**	**-45**	**-70**	**7**
			5.02	-51	-58	4
			3.88	-48	-73	-5
Left Fusiform Gyrus	21	.425	5.73	-33	-58	-20
**Left Precentral Gyrus**	**116**	**.002**	**5.56**	**-48**	**-4**	**37**
			5.29	-36	-1	7
			4.91	-36	-1	40
Left Cuneus	41	.118	5.39	-18	-76	34
			4.14	-18	-58	28
Left IFG	10	.786	4.75	-54	14	10
??	23	.374	4.64	9	-13	-11
			3.79	-3	-22	-14
Left Middle Occipital Gyrus	13	.679	4.63	-15	-97	1
Right Cerebellum	15	.608	4.14	39	-49	-32
Right MTG	18	.511	4,13	60	-58	4
			4.00	45	-46	-2
			3.84	51	-52	-2
**SYNCHRONY*GAZE [Synchronous (facing Toward > Facing Away) > Asynchronous (Facing Toward > Facing Away)]**						
No suprathreshold clusters						
**(When all parameters are included in the model) INCREASED LIKING**						
Left Angular Gyrus/IPL		.473	4.36	-42	-61	43
**INCREASED TOGETHERNESS**						
Left superior temporal gyrus/IPL		.792	3.87	-60	-40	22

Supplementary Table 4a. Action observation network ROIs, linear mixed models (percent signal change)

	**left IFG**				**left IPL**				**left SI**				**left SMA**			
Predictors	*Estimates*	*CI*	*Statistic*	*p*	*Estimates*	*CI*	*Statistic*	*p*	*Estimates*	*CI*	*Statistic*	*p*	*Estimates*	*CI*	*Statistic*	*p*
(Intercept)	0.18	-0.56, 0.92	0.48	0.633	-0.04	-0.45, 0.37	-0.21	0.834	0.50	0.17, 0.84	2.99	**0.004**	0.45	-0.05, 0.94	1.79	0.077
age.c	0.45	-0.30, 1.20	1.20	0.233	0.07	-0.35, 0.49	0.33	0.740	0.17	-0.17, 0.51	1.01	0.316	-0.02	-0.52, 0.49	-0.06	0.951
dance.c	-0.23	-0.98, 0.53	-0.60	0.552	-0.16	-0.58, 0.25	-0.78	0.436	-0.10	-0.44, 0.24	-0.57	0.570	-0.19	-0.70, 0.31	-0.77	0.444
gaze.c	0.14	0.03, 0.26	2.51	**0.014**	0.21	0.12, 0.31	4.37	**<0.001**	0.17	0.09, 0.26	4.03	**<0.001**	0.06	-0.05, 0.16	1.07	0.288
sync.c	-0.11	-0.22, 0.01	-1.84	0.070	0.12	0.02, 0.22	2.43	**0.018**	0.05	-0.04, 0.13	1.09	0.281	-0.11	-0.22, -0.01	-2.10	**0.039**
sync.c:gaze.c	0.26	0.03, 0.49	2.25	**0.028**	0.07	-0.12, 0.27	0.73	0.465	0.01	-0.17, 0.18	0.09	0.930	0.06	-0.16, 0.27	0.52	0.605
**Random Effects**																
σ^2	0.07				0.05				0.04				0.06			
τ_{00}	2.74 $_{sid}$				0.83 $_{sid}$				0.56 $_{sid}$				1.23 $_{sid}$			
ICC	0.98				0.95				0.94				0.96			
N	20 $_{sid}$				20 $_{sid}$				20 $_{sid}$				20 $_{sid}$			
Observations	80				80				80				80			
Marginal R^2 / Conditional R^2	0.079 / 0.979				0.048 / 0.948				0.068 / 0.941				0.033 / 0.957			

Supplementary Table 4b. Action observation network ROIs, linear mixed models (percent signal change)

	left STS				left FG				left lateral occipital				left dPMC			
Predictors	*Estimates*	*CI*	*Statistic*	*p*	*Estimates*	*CI*	*Statistic*	*p*	*Estimates*	*CI*	*Statistic*	*p*	*Estimates*	*CI*	*Statistic*	*p*
(Intercept)	0.93	0.40, 1.45	3.53	**0.001**	0.74	0.17, 1.30	2.59	**0.011**	2.50	1.91, 3.08	8.49	**<0.001**	0.28	-0.03, 0.60	1.81	0.075
age.c	0.26	-0.28, 0.79	0.96	0.339	0.44	-0.13, 1.02	1.53	0.129	0.23	-0.37, 0.82	0.76	0.451	0.07	-0.25, 0.39	0.43	0.671
dance.c	-0.09	-0.63, 0.44	-0.35	0.725	-0.24	-0.81, 0.34	-0.82	0.416	-0.18	-0.78, 0.41	-0.62	0.539	0.02	-0.29, 0.34	0.15	0.880
gaze.c	0.15	0.06, 0.25	3.28	**0.002**	0.08	-0.01, 0.17	1.71	0.092	0.20	0.09, 0.31	3.71	**<0.001**	0.10	0.03, 0.17	2.78	**0.007**
sync.c	-0.07	-0.17, 0.02	-1.58	0.118	0.07	-0.02, 0.16	1.54	0.128	0.00	-0.10, 0.11	0.08	0.937	-0.01	-0.09, 0.06	-0.41	0.686
sync.c:gaze.c	0.09	-0.10, 0.27	0.91	0.366	0.01	-0.17, 0.19	0.10	0.919	0.00	-0.22, 0.22	0.00	0.999	-0.02	-0.16, 0.12	-0.26	0.798
**Random Effects**																
σ^2	0.04				0.04				0.06				0.03			
τ_{00}	1.37 $_{sid}$				1.60 $_{sid}$				1.71 $_{sid}$				0.49 $_{sid}$			
ICC	0.97				0.98				0.97				0.95			
N	20 $_{sid}$				20 $_{sid}$				20 $_{sid}$				20 $_{sid}$			
Observations	80				80				80				80			
Marginal R^2 / Conditional R^2	0.051 / 0.970				0.121 / 0.978				0.045 / 0.968				0.016 / 0.951			

Supplementary Table 4c. Action observation network ROIs, linear mixed models (percent signal change)

	right IFG2				right IFG				right IPL				right SI				right SMA			
Predictors	*Estimates*	*CI*	*Statistic*	*p*	*Estimates*	*CI*	*Statistic*	*p*	*Estimates*	*CI*	*Statistic*	*p*	*Estimates*	*CI*	*Statistic*	*p*	*Estimates*	*CI*	*Statistic*	*p*
(Intercept)	0.46	-0.16, 1.08	1.48	0.144	0.95	0.52, 1.37	4.43	**<0.001**	0.60	0.22, 0.98	3.15	**0.002**	0.06	-0.32, 0.43	0.29	0.773	0.33	-0.24, 0.91	1.16	0.248
age.c	-0.14	-0.77, 0.49	-0.44	0.663	0.25	-0.18, 0.69	1.18	0.243	0.13	-0.25, 0.52	0.69	0.491	0.11	-0.27, 0.50	0.58	0.565	-0.20	-0.78, 0.38	-0.69	0.493
dance.c	-0.39	-1.02, 0.25	-1.22	0.228	-0.09	-0.52, 0.34	-0.41	0.681	-0.12	-0.51, 0.26	-0.63	0.530	0.01	-0.37, 0.40	0.06	0.951	-0.21	-0.79, 0.37	-0.73	0.466
gaze.c	0.08	-0.02, 0.18	1.61	0.111	0.06	-0.05, 0.17	1.09	0.277	0.12	0.02, 0.22	2.32	**0.023**	0.16	0.06, 0.25	3.38	**0.001**	0.06	-0.05, 0.17	1.12	0.265
sync.c	0.02	-0.08, 0.12	0.36	0.721	0.03	-0.09, 0.14	0.46	0.650	0.04	-0.06, 0.14	0.81	0.423	0.19	0.10, 0.28	4.05	**<0.001**	-0.08	-0.19, 0.03	-1.49	0.140
sync.c:gaze.c	0.11	-0.10, 0.31	1.04	0.301	0.04	-0.18, 0.26	0.35	0.725	-0.06	-0.26, 0.14	-0.56	0.574	0.07	-0.12, 0.25	0.74	0.460	0.01	-0.20, 0.23	0.12	0.901
**Random Effects**																				
σ^2	0.05				0.06				0.05				0.04				0.06			
τ_{00}	1.94 sid				0.89 sid				0.72 sid				0.72 sid				1.63 sid			
ICC	0.97				0.94				0.93				0.94				0.96			
N	20 sid				20 sid				20 sid				20 sid				20 sid			
Observations	80				80				80				80				80			
Marginal R^2 / Conditional R^2	0.085 / 0.976				0.066 / 0.940				0.041 / 0.936				0.036 / 0.946				0.056 / 0.967			

Supplementary Table 4d. Action observation network ROIs, linear mixed models (percent signal change).

	right IPS				right SPL				right STS				right FG				right lateral occipital				right dPMC			
Predictors	*Estimates*	*CI*	*Statistic*	*p*	*Estimates*	*CI*	*Statistic*	*p*	*Estimates*	*CI*	*Statistic*	*p*	*Estimates*	*CI*	*Statistic*	*p*	*Estimates*	*CI*	*Statistic*	*p*	*Estimates*	*CI*	*Statistic*	*p*
(Intercept)	0.78	0.31, 1.24	3.34	**0.001**	0.15	-0.19, 0.49	0.87	0.389	1.22	0.87, 1.57	6.89	**<0.001**	1.84	1.42, 2.27	8.61	**<0.001**	3.30	2.54, 4.06	8.63	**<0.001**	0.39	0.02, 0.76	2.10	**0.040**
age.c	0.27	-0.20, 0.75	1.16	0.248	0.32	-0.03, 0.67	1.80	0.075	0.41	0.05, 0.76	2.26	**0.027**	0.00	-0.43, 0.44	0.02	0.986	0.57	-0.20, 1.35	1.48	0.144	0.07	-0.30, 0.45	0.39	0.696
dance.c	-0.18	-0.65, 0.29	-0.77	0.442	-0.34	-0.69, 0.01	-1.92	0.058	-0.20	-0.56, 0.16	-1.10	0.276	-0.19	-0.62, 0.25	-0.86	0.393	-0.37	-1.15, 0.40	-0.96	0.340	-0.12	-0.50, 0.26	-0.64	0.525
gaze.c	0.07	-0.03, 0.17	1.49	0.140	0.11	0.01, 0.20	2.20	**0.031**	0.13	0.04, 0.23	2.85	**0.006**	0.07	-0.05, 0.19	1.17	0.246	0.16	0.01, 0.31	2.19	**0.032**	0.09	0.00, 0.17	2.05	**0.044**
sync.c	-0.01	-0.11, 0.09	-0.23	0.817	0.02	-0.07, 0.12	0.49	0.623	-0.15	-0.24, -0.06	-3.22	**0.002**	0.08	-0.04, 0.19	1.29	0.201	0.00	-0.14, 0.15	0.06	0.952	-0.03	-0.11, 0.06	-0.61	0.544
sync.c:gaze.c	-0.01	-0.21, 0.19	-0.12	0.907	-0.07	-0.26, 0.12	-0.72	0.477	-0.06	-0.25, 0.13	-0.63	0.533	0.08	-0.16, 0.31	0.66	0.510	-0.10	-0.39, 0.20	-0.66	0.513	-0.08	-0.25, 0.08	-0.99	0.327
**Random Effects**																								
σ^2	0.05				0.05				0.04				0.07				0.11				0.03			
τ_{00}	1.07 $_{sid}$				0.58 $_{sid}$				0.62 $_{sid}$				0.90 $_{sid}$				2.90 $_{sid}$				0.68 $_{sid}$			
ICC	0.96				0.93				0.93				0.93				0.96				0.95			
N	20 $_{sid}$				20 $_{sid}$				20 $_{sid}$				20 $_{sid}$				20 $_{sid}$				20 $_{sid}$			
Observations	80				80				80				80				80				80			
Marginal R^2 / Conditional R^2	0.080 / 0.959				0.230 / 0.943				0.227 / 0.949				0.037 / 0.932				0.122 / 0.969				0.027 / 0.953			

Supplementary Table 5a. Theory of Mind Network ROIs, linear mixed models (percent signal change).

	dMPFC				left STS				left TPJ				left TP			
Predictors	*Estimates*	*CI*	*Statistic*	*p*	*Estimates*	*CI*	*Statistic*	*p*	*Estimates*	*CI*	*Statistic*	*p*	*Estimates*	*CI*	*Statistic*	*p*
(Intercept)	-1.04	-1.54, -0.55	-4.19	**<0.001**	0.17	-0.29, 0.63	0.74	0.465	-0.10	-0.55, 0.36	-0.41	0.680	0.74	0.21, 1.26	2.79	**0.007**
age.c	-0.18	-0.69, 0.32	-0.73	0.466	0.04	-0.42, 0.51	0.19	0.850	-0.03	-0.49, 0.44	-0.11	0.911	-0.21	-0.74, 0.33	-0.78	0.439
dance.c	0.17	-0.33, 0.67	0.67	0.503	0.05	-0.42, 0.52	0.23	0.821	0.23	-0.23, 0.70	0.99	0.323	0.33	-0.20, 0.86	1.23	0.222
gaze.c	0.08	-0.09, 0.25	0.94	0.352	0.01	-0.09, 0.11	0.25	0.805	0.10	-0.00, 0.21	1.93	0.057	-0.04	-0.17, 0.08	-0.66	0.512
sync.c	0.17	0.00, 0.33	2.00	**0.049**	0.04	-0.06, 0.14	0.86	0.392	0.08	-0.02, 0.19	1.59	0.116	0.02	-0.11, 0.14	0.28	0.784
sync.c:gaze.c	0.27	-0.07, 0.60	1.59	0.116	0.08	-0.12, 0.28	0.78	0.438	0.04	-0.17, 0.25	0.40	0.694	0.07	-0.18, 0.33	0.58	0.562
**Random Effects**																
σ^2	0.14				0.05				0.06				0.08			
τ_{00}	1.20 $_{sid}$				1.07 $_{sid}$				1.05 $_{sid}$				1.37 $_{sid}$			
ICC	0.90				0.96				0.95				0.94			
N	20 $_{sid}$				20 $_{sid}$				20 $_{sid}$				20 $_{sid}$			
Observations	80				80				80				80			
Marginal R^2 / Conditional R^2	0.048 / 0.901				0.006 / 0.956				0.050 / 0.951				0.085 / 0.949			

Supplementary Table 5b. Theory of Mind Network ROIs, linear mixed models (percent signal change).

	precunues				right STS				right TPJ				right TP				vMPFC			
Predictors	*Estimates*	*CI*	*Statistic*	*p*	*Estimates*	*CI*	*Statistic*	*p*	*Estimates*	*CI*	*Statistic*	*p*	*Estimates*	*CI*	*Statistic*	*p*	*Estimates*	*CI*	*Statistic*	*p*
(Intercept)	-0.95	-1.42, -0.48	-4.01	**<0.001**	0.51	0.15, 0.86	2.86	**0.006**	0.01	-0.53, 0.55	0.05	0.959	0.42	-0.11, 0.94	1.59	0.116	-1.97	-2.96, -0.98	-3.97	**<0.001**
age.c	0.27	-0.21, 0.75	1.12	0.267	-0.18	-0.53, 0.18	-0.98	0.331	0.05	-0.50, 0.60	0.17	0.864	0.17	-0.36, 0.70	0.63	0.531	-0.47	-1.48, 0.53	-0.94	0.349
dance.c	0.06	-0.41, 0.54	0.27	0.789	-0.06	-0.42, 0.30	-0.33	0.739	0.18	-0.37, 0.73	0.67	0.508	-0.00	-0.53, 0.53	-0.00	0.997	0.03	-0.97, 1.04	0.07	0.947
gaze.c	0.04	-0.12, 0.20	0.49	0.623	0.11	0.01, 0.20	2.29	**0.025**	0.08	-0.01, 0.18	1.73	0.088	-0.03	-0.15, 0.08	-0.57	0.568	0.08	-0.17, 0.33	0.63	0.529
sync.c	0.01	-0.14, 0.17	0.19	0.851	-0.02	-0.11, 0.08	-0.36	0.719	-0.14	-0.24, -0.05	-2.96	**0.004**	0.04	-0.08, 0.15	0.62	0.537	0.25	0.00, 0.50	2.01	**0.048**
sync.c:gaze.c	0.07	-0.24, 0.39	0.46	0.645	-0.07	-0.26, 0.11	-0.79	0.432	-0.03	-0.22, 0.16	-0.30	0.766	-0.09	-0.32, 0.14	-0.77	0.444	-0.19	-0.70, 0.31	-0.77	0.444
**Random Effects**																				
σ^2	0.13				0.04				0.05				0.07				0.32			
τ_{00}	1.09 $_{sid}$				0.61 $_{sid}$				1.46 $_{sid}$				1.37 $_{sid}$				4.84 $_{sid}$			
ICC	0.90				0.93				0.97				0.95				0.94			
N	20 $_{sid}$				20 $_{sid}$				20 $_{sid}$				20 $_{sid}$				20 $_{sid}$			
Observations	80				80				80				80				80			
Marginal R^2 / Conditional R^2	0.063 / 0.902				0.058 / 0.936				0.029 / 0.970				0.020 / 0.955				0.045 / 0.941			

Supplementary Table 6a. Linear mixed models for AON responses with liking x synchrony x gaze direction included as fixed effects in the model.

	left IFG				left IPL				left SI				left SMA			
Predictors	*Estimates*	*CI*	*Statistic*	*p*	*Estimates*	*CI*	*Statistic*	*p*	*Estimates*	*CI*	*Statistic*	*p*	*Estimates*	*CI*	*Statistic*	*p*
(Intercept)	0.16	-0.58, 0.89	0.42	0.675	-0.05	-0.45, 0.35	-0.26	0.793	0.49	0.15, 0.82	2.91	**0.005**	0.44	-0.05, 0.92	1.81	0.075
gaze.c	0.14	-0.01, 0.30	1.86	0.067	0.26	0.13, 0.39	4.00	**<0.001**	0.19	0.07, 0.31	3.22	**0.002**	0.09	-0.05, 0.23	1.29	0.202
liking.c	0.06	-0.05, 0.17	1.06	0.293	0.02	-0.07, 0.11	0.43	0.669	0.01	-0.07, 0.09	0.23	0.821	0.02	-0.08, 0.12	0.34	0.733
liking.c:gaze.c	0.10	-0.06, 0.26	1.21	0.232	0.09	-0.05, 0.22	1.26	0.213	0.07	-0.05, 0.19	1.17	0.248	0.16	0.01, 0.31	2.20	**0.031**
liking.c:sync.c	0.05	-0.13, 0.23	0.56	0.576	0.00	-0.15, 0.16	0.05	0.963	0.03	-0.10, 0.17	0.47	0.638	-0.01	-0.17, 0.16	-0.10	0.923
liking.c:sync.c:gaze.c	-0.04	-0.35, 0.27	-0.26	0.793	-0.18	-0.44, 0.09	-1.33	0.186	-0.05	-0.29, 0.18	-0.45	0.655	-0.13	-0.41, 0.16	-0.89	0.377
sync.c	-0.17	-0.35, 0.01	-1.85	0.069	0.11	-0.04, 0.27	1.46	0.150	0.04	-0.09, 0.18	0.64	0.527	-0.11	-0.28, 0.05	-1.38	0.171
sync.c:gaze.c	0.14	-0.17, 0.45	0.91	0.366	-0.03	-0.29, 0.23	-0.22	0.830	-0.09	-0.32, 0.15	-0.73	0.470	-0.13	-0.42, 0.15	-0.95	0.343
**Random Effects**																
σ^2	0.07				0.05				0.04				0.06			
τ_{00}	2.69 $_{sid}$				0.78 $_{sid}$				0.54 $_{sid}$				1.14 $_{sid}$			
ICC	0.98				0.94				0.93				0.95			
N	20 $_{sid}$				20 $_{sid}$				20 $_{sid}$				20 $_{sid}$			
Observations	80				80				80				80			
Marginal R^2 / Conditional R^2	0.006 / 0.976				0.022 / 0.944				0.016 / 0.934				0.008 / 0.954			

Supplementary Table 6b. Linear mixed models for AON responses with liking x synchrony x gaze direction included as fixed effects in the model.

	**left STS**				**left FG**				**left lateral occipital**				**left dPMC**			
Predictors	*Estimates*	*CI*	*Statistic*	*p*	*Estimates*	*CI*	*Statistic*	*p*	*Estimates*	*CI*	*Statistic*	*p*	*Estimates*	*CI*	*Statistic*	*p*
(Intercept)	0.93	0.42, 1.44	3.61	**0.001**	0.72	0.14, 1.31	2.49	**0.015**	2.46	1.89, 3.03	8.57	**<0.001**	0.28	-0.02, 0.58	1.84	0.069
gaze.c	0.20	0.08, 0.33	3.21	**0.002**	0.11	-0.01, 0.23	1.76	0.082	0.24	0.09, 0.38	3.20	**0.002**	0.10	0.00, 0.19	2.08	**0.041**
liking.c	-0.02	-0.11, 0.07	-0.41	0.686	0.04	-0.05, 0.13	0.86	0.391	0.02	-0.09, 0.12	0.36	0.717	0.02	-0.05, 0.09	0.60	0.551
liking.c:gaze.c	0.08	-0.05, 0.21	1.26	0.212	0.02	-0.11, 0.15	0.34	0.738	0.07	-0.09, 0.22	0.85	0.399	0.09	-0.00, 0.19	1.90	0.061
liking.c:sync.c	-0.03	-0.18, 0.11	-0.45	0.654	0.03	-0.12, 0.17	0.35	0.727	0.09	-0.08, 0.27	1.10	0.275	-0.01	-0.12, 0.10	-0.15	0.885
liking.c:sync.c:gaze.c	-0.15	-0.41, 0.10	-1.21	0.232	-0.13	-0.38, 0.12	-1.05	0.298	-0.11	-0.41, 0.19	-0.74	0.462	-0.02	-0.21, 0.17	-0.20	0.844
sync.c	-0.04	-0.18, 0.11	-0.49	0.628	0.03	-0.11, 0.18	0.46	0.648	-0.01	-0.18, 0.16	-0.09	0.932	-0.03	-0.14, 0.08	-0.57	0.573
sync.c:gaze.c	-0.01	-0.27, 0.24	-0.10	0.921	-0.01	-0.26, 0.23	-0.11	0.916	-0.10	-0.39, 0.20	-0.67	0.505	-0.13	-0.32, 0.06	-1.32	0.190
**Random Effects**																
σ^2	0.04				0.04				0.06				0.02			
τ_{00}	1.30 $_{sid}$				1.68 $_{sid}$				1.62 $_{sid}$				0.45 $_{sid}$			
ICC	0.97				0.98				0.96				0.95			
N	20 $_{sid}$				20 $_{sid}$				20 $_{sid}$				20 $_{sid}$			
Observations	80				80				80				80			
Marginal R^2 / Conditional R^2	0.008 / 0.967				0.003 / 0.976				0.007 / 0.965				0.009 / 0.948			

Supplementary Table 6c. Linear mixed models for AON responses with liking x synchrony x gaze direction included as fixed effects in the model.

	**right IFG2**				**right IFG**				**right IPL**				**right SI**				**right SMA**			
Predictors	*Estimates*	*CI*	*Statistic*	*p*	*Estimates*	*CI*	*Statistic*	*p*	*Estimates*	*CI*	*Statistic*	*p*	*Estimates*	*CI*	*Statistic*	*p*	*Estimates*	*CI*	*Statistic*	*p*
(Intercept)	0.46	-0.16, 1.09	1.47	0.146	0.94	0.51, 1.37	4.39	**<0.001**	0.59	0.22, 0.96	3.14	**0.002**	0.06	-0.31, 0.43	0.33	0.742	0.33	-0.23, 0.90	1.17	0.246
gaze.c	0.07	-0.07, 0.22	1.06	0.292	0.01	-0.14, 0.16	0.17	0.869	0.16	0.02, 0.30	2.23	**0.029**	0.18	0.05, 0.31	2.86	**0.006**	0.09	-0.06, 0.23	1.19	0.236
liking.c	0.05	-0.05, 0.15	1.03	0.307	0.06	-0.05, 0.17	1.13	0.262	0.02	-0.08, 0.11	0.31	0.754	-0.01	-0.10, 0.09	-0.12	0.908	0.04	-0.06, 0.14	0.76	0.450
liking.c:gaze.c	0.02	-0.13, 0.16	0.21	0.831	0.07	-0.08, 0.23	0.93	0.356	0.02	-0.13, 0.16	0.26	0.792	0.04	-0.09, 0.17	0.63	0.532	0.13	-0.02, 0.28	1.75	0.084
liking.c:sync.c	0.00	-0.16, 0.16	0.01	0.992	0.00	-0.17, 0.18	0.05	0.957	0.03	-0.13, 0.20	0.41	0.683	-0.03	-0.18, 0.12	-0.43	0.667	-0.03	-0.19, 0.14	-0.30	0.765
liking.c:sync.c:gaze.c	-0.02	-0.30, 0.26	-0.14	0.892	0.11	-0.20, 0.41	0.71	0.480	-0.13	-0.41, 0.15	-0.94	0.348	-0.09	-0.34, 0.17	-0.68	0.498	-0.12	-0.41, 0.17	-0.84	0.406
sync.c	-0.04	-0.21, 0.12	-0.52	0.607	-0.05	-0.23, 0.13	-0.58	0.567	0.03	-0.13, 0.19	0.39	0.697	0.20	0.05, 0.35	2.72	**0.008**	-0.11	-0.28, 0.06	-1.33	0.189
sync.c:gaze.c	0.10	-0.18, 0.38	0.72	0.473	-0.03	-0.34, 0.27	-0.23	0.821	-0.09	-0.36, 0.19	-0.61	0.545	0.02	-0.23, 0.28	0.19	0.848	-0.13	-0.42, 0.16	-0.90	0.371
**Random Effects**																				
σ^2	0.05				0.06				0.05				0.04				0.06			
τ_{00}	1.94 $_{sid}$				0.89 $_{sid}$				0.68 $_{sid}$				0.65 $_{sid}$				1.58 $_{sid}$			
ICC	0.97				0.93				0.93				0.94				0.96			
N	20 $_{sid}$				20 $_{sid}$				20 $_{sid}$				20 $_{sid}$				20 $_{sid}$			
Observations	80				80				80				80				80			
Marginal R^2 / Conditional R^2	0.002 / 0.973				0.005 / 0.934				0.007 / 0.927				0.023 / 0.937				0.005 / 0.965			

Supplementary Table 6d. Linear mixed models for AON responses with liking x synchrony x gaze direction included as fixed effects in the model.

	**right IPS**				**right SPL**				**right STS**				**right FG**				**right lateral occipital**				**right dPMC**			
Predictors	*Est.*	*CI*	*Stat.*	*p*	*Est.*	*CI*	*Stat.*	*p*	*Est.*	*CI*	*Stat.*	*p*	*Est.*	*CI*	*Stat.*	*p*	*Est.*	*CI*	*Stat.*	*p*	*Est.*	*CI*	*Stat.*	*p*
(Intercept)	0.79	0.32, 1.26	3.38	**0.001**	0.14	-0.24, 0.53	0.75	0.457	1.21	0.82, 1.60	6.19	**<0.001**	1.85	1.43, 2.27	8.76	**<0.001**	3.30	2.52, 4.08	8.39	**<0.001**	0.38	0.02, 0.74	2.11	**0.038**
gaze.c	0.10	-0.04, 0.24	1.45	0.152	0.14	0.00, 0.27	2.03	**0.046**	0.18	0.06, 0.31	2.94	**0.004**	0.09	-0.07, 0.24	1.13	0.262	0.21	0.00, 0.41	2.02	**0.047**	0.10	-0.02, 0.21	1.71	0.091
liking.c	-0.00	-0.10, 0.09	-0.06	0.953	-0.01	-0.10, 0.09	-0.17	0.862	0.00	-0.09, 0.09	0.01	0.995	0.09	-0.02, 0.20	1.58	0.118	0.01	-0.14, 0.15	0.12	0.906	0.01	-0.07, 0.09	0.17	0.868
liking.c:gaze.c	0.04	-0.11, 0.18	0.51	0.609	0.00	-0.14, 0.14	0.05	0.961	0.11	-0.02, 0.24	1.65	0.103	0.03	-0.13, 0.19	0.36	0.719	0.05	-0.16, 0.26	0.44	0.659	0.11	-0.01, 0.22	1.80	0.075
liking.c:sync.c	-0.06	-0.22, 0.10	-0.73	0.465	0.01	-0.14, 0.17	0.19	0.853	-0.00	-0.15, 0.14	-0.00	0.997	-0.04	-0.22, 0.14	-0.44	0.663	-0.02	-0.25, 0.22	-0.14	0.893	0.00	-0.13, 0.13	0.05	0.961
liking.c:sync.c:gaze.c	-0.09	-0.37, 0.18	-0.67	0.504	-0.09	-0.36, 0.18	-0.65	0.518	-0.17	-0.42, 0.09	-1.32	0.192	-0.15	-0.46, 0.17	-0.94	0.348	-0.16	-0.56, 0.25	-0.77	0.443	-0.04	-0.26, 0.18	-0.36	0.722
sync.c	0.00	-0.16, 0.16	0.01	0.995	0.04	-0.12, 0.20	0.51	0.612	-0.13	-0.28, 0.01	-1.82	0.073	-0.02	-0.20, 0.16	-0.22	0.829	0.01	-0.23, 0.24	0.06	0.950	-0.02	-0.15, 0.11	-0.36	0.720
sync.c:gaze.c	-0.04	-0.32, 0.23	-0.31	0.760	-0.08	-0.35, 0.19	-0.59	0.556	-0.19	-0.44, 0.06	-1.52	0.133	0.08	-0.24, 0.39	0.49	0.626	-0.15	-0.55, 0.26	-0.73	0.471	-0.21	-0.43, 0.01	-1.87	0.066
**Random Effects**																								
σ^2	0.05				0.05				0.04				0.07				0.11				0.03			
τ_{00}	1.07 $_{sid}$				0.71 $_{sid}$				0.74 $_{sid}$				0.86 $_{sid}$				3.03 $_{sid}$				0.64 $_{sid}$			
ICC	0.95				0.94				0.95				0.93				0.96				0.95			
N	20 $_{sid}$				20 $_{sid}$				20 $_{sid}$				20 $_{sid}$				20 $_{sid}$				20 $_{sid}$			
Observations	80				80				80				80				80				80			
Marginal R^2 / Conditional R^2	0.002 / 0.953				0.005 / 0.935				0.017 / 0.946				0.010 / 0.928				0.003 / 0.964				0.006 / 0.949			

Supplementary Table 7a. Linear mixed models for AON responses with togetherness x synchrony x gaze direction included as fixed effects in the model.

	left IFG				left IPL				left SI				left SMA			
Predictors	*Estimates*	*CI*	*Statistic*	*p*	*Estimates*	*CI*	*Statistic*	*p*	*Estimates*	*CI*	*Statistic*	*p*	*Estimates*	*CI*	*Statistic*	*p*
(Intercept)	0.26	-0.47, 0.99	0.70	0.484	0.02	-0.37, 0.41	0.10	0.923	0.56	0.23, 0.89	3.36	**0.001**	0.50	0.03, 0.98	2.11	**0.038**
gaze.c	0.24	0.03, 0.46	2.26	**0.027**	0.20	0.02, 0.39	2.18	**0.033**	0.21	0.04, 0.37	2.46	**0.016**	0.09	-0.12, 0.29	0.83	0.409
sync.c	-0.08	-0.34, 0.18	-0.60	0.548	0.01	-0.21, 0.24	0.11	0.910	0.01	-0.19, 0.22	0.14	0.890	-0.18	-0.43, 0.07	-1.45	0.152
sync.c:gaze.c	0.44	0.06, 0.83	2.32	**0.023**	0.17	-0.16, 0.49	1.02	0.310	0.16	-0.14, 0.45	1.07	0.289	0.22	-0.14, 0.59	1.22	0.226
togetherness.c	0.01	-0.14, 0.16	0.15	0.883	0.09	-0.04, 0.21	1.32	0.192	0.04	-0.08, 0.15	0.66	0.511	0.07	-0.08, 0.21	0.94	0.349
togetherness.c:gaze.c	-0.05	-0.24, 0.13	-0.57	0.571	-0.00	-0.16, 0.16	-0.04	0.969	-0.06	-0.20, 0.09	-0.81	0.420	-0.07	-0.25, 0.11	-0.79	0.434
togetherness.c:sync.c	-0.21	-0.41, -0.01	-2.06	**0.043**	-0.18	-0.35, -0.00	-2.03	**0.046**	-0.13	-0.29, 0.03	-1.66	0.102	-0.13	-0.33, 0.06	-1.39	0.169
togetherness.c:sync.c:gaze.c	-0.30	-0.69, 0.09	-1.55	0.127	-0.09	-0.42, 0.24	-0.54	0.591	-0.14	-0.44, 0.15	-0.96	0.341	-0.18	-0.55, 0.19	-0.99	0.328
**Random Effects**																
σ^2	0.06				0.05				0.04				0.06			
τ_{00}	2.66 $_{sid}$				0.74 $_{sid}$				0.52 $_{sid}$				1.10 $_{sid}$			
ICC	0.98				0.94				0.93				0.95			
N	20 $_{sid}$				20 $_{sid}$				20 $_{sid}$				20 $_{sid}$			
Observations	80				80				80				80			
Marginal R^2 / Conditional R^2	0.007 / 0.977				0.029 / 0.943				0.020 / 0.934				0.009 / 0.951			

Supplementary Table 7b. Linear mixed models for AON responses with togetherness x synchrony x gaze direction included as fixed effects in the model.

	left STS				left FG				left lateral occipital				left dPMC			
Predictors	*Estimates*	*CI*	*Statistic*	*p*	*Estimates*	*CI*	*Statistic*	*p*	*Estimates*	*CI*	*Statistic*	*p*	*Estimates*	*CI*	*Statistic*	*p*
(Intercept)	0.97	0.46, 1.48	3.77	**<0.001**	0.78	0.20, 1.35	2.69	**0.009**	2.57	2.01, 3.14	9.03	**<0.001**	0.34	0.04, 0.65	2.28	**0.026**
gaze.c	0.14	-0.04, 0.32	1.52	0.133	0.09	-0.09, 0.26	0.97	0.333	0.09	-0.11, 0.29	0.94	0.349	0.08	-0.05, 0.21	1.19	0.240
sync.c	-0.17	-0.39, 0.06	-1.49	0.141	0.00	-0.21, 0.22	0.04	0.967	-0.20	-0.44, 0.04	-1.65	0.103	-0.07	-0.23, 0.09	-0.86	0.394
sync.c:gaze.c	0.23	-0.09, 0.55	1.42	0.161	0.15	-0.16, 0.46	0.97	0.337	0.15	-0.20, 0.51	0.87	0.388	0.13	-0.11, 0.37	1.09	0.278
togetherness.c	0.07	-0.05, 0.20	1.13	0.260	0.06	-0.06, 0.18	0.95	0.347	0.14	-0.00, 0.28	1.97	0.053	0.04	-0.05, 0.13	0.86	0.391
togetherness.c:gaze.c	-0.07	-0.23, 0.09	-0.92	0.359	-0.07	-0.22, 0.08	-0.91	0.366	-0.03	-0.21, 0.14	-0.37	0.709	-0.05	-0.16, 0.07	-0.82	0.413
togetherness.c:sync.c	-0.08	-0.25, 0.09	-0.98	0.331	-0.09	-0.25, 0.08	-1.06	0.293	-0.21	-0.40, -0.03	-2.27	**0.026**	-0.15	-0.28, -0.03	-2.44	**0.017**
togetherness.c:sync.c:gaze.c	-0.07	-0.39, 0.26	-0.42	0.679	-0.11	-0.43, 0.20	-0.71	0.477	0.10	-0.26, 0.46	0.55	0.585	-0.00	-0.24, 0.24	-0.02	0.987
**Random Effects**																
σ^2	0.04				0.04				0.05				0.02			
τ_{00}	1.27 $_{sid}$				1.63 $_{sid}$				1.58 $_{sid}$				0.44 $_{sid}$			
ICC	0.97				0.98				0.97				0.95			
N	20 $_{sid}$				20 $_{sid}$				20 $_{sid}$				20 $_{sid}$			
Observations	80				80				80				80			
Marginal R^2 / Conditional R^2	0.009 / 0.966				0.004 / 0.975				0.014 / 0.968				0.012 / 0.948			

Supplementary Table 7c. Linear mixed models for AON responses with togetherness x synchrony x gaze direction included as fixed effects in the model.

	right IFG2				right IFG				right IPL				right SI				right SMA			
Predictors	*Estimates*	*CI*	*Statistic*	*p*	*Estimates*	*CI*	*Statistic*	*p*	*Estimates*	*CI*	*Statistic*	*p*	*Estimates*	*CI*	*Statistic*	*p*	*Estimates*	*CI*	*Statistic*	*p*
(Intercept)	0.48	-0.14, 1.10	1.53	0.130	1.05	0.63, 1.48	4.95	**<0.001**	0.70	0.32, 1.07	3.70	**<0.001**	0.09	-0.27, 0.46	0.52	0.608	0.40	-0.17, 0.96	1.40	0.165
gaze.c	0.12	-0.08, 0.31	1.17	0.246	0.12	-0.09, 0.32	1.15	0.253	0.15	-0.03, 0.34	1.63	0.108	0.16	-0.02, 0.35	1.81	0.075	0.11	-0.10, 0.32	1.06	0.293
sync.c	-0.06	-0.30, 0.18	-0.50	0.620	0.00	-0.25, 0.25	0.00	0.999	0.05	-0.17, 0.28	0.48	0.634	0.14	-0.08, 0.36	1.29	0.201	-0.13	-0.38, 0.12	-1.06	0.292
sync.c:gaze.c	0.20	-0.15, 0.55	1.16	0.252	0.34	-0.02, 0.70	1.87	0.066	0.17	-0.16, 0.51	1.02	0.312	0.18	-0.14, 0.50	1.10	0.276	0.11	-0.26, 0.47	0.58	0.564
togetherness.c	0.08	-0.06, 0.21	1.12	0.265	0.04	-0.10, 0.18	0.57	0.573	0.00	-0.13, 0.13	0.00	0.999	0.04	-0.08, 0.17	0.65	0.517	0.06	-0.09, 0.20	0.81	0.420
togetherness.c:gaze.c	-0.06	-0.23, 0.11	-0.74	0.463	-0.12	-0.29, 0.06	-1.31	0.196	-0.07	-0.23, 0.10	-0.84	0.404	-0.04	-0.20, 0.12	-0.53	0.600	0.00	-0.18, 0.18	0.01	0.991
togetherness.c:sync.c	-0.02	-0.20, 0.16	-0.21	0.834	-0.26	-0.45, -0.07	-2.68	**0.009**	-0.24	-0.42, -0.07	-2.72	**0.008**	-0.10	-0.27, 0.07	-1.13	0.261	-0.18	-0.37, 0.01	-1.84	0.070
togetherness.c:sync.c:gaze.c	-0.21	-0.56, 0.14	-1.21	0.229	-0.22	-0.59, 0.15	-1.19	0.239	-0.10	-0.44, 0.24	-0.57	0.568	-0.08	-0.41, 0.24	-0.52	0.608	-0.22	-0.59, 0.15	-1.20	0.234
**Random Effects**																				
σ^2	0.05				0.06				0.05				0.04				0.06			
τ_{00}	1.91 $_{sid}$				0.86 $_{sid}$				0.67 $_{sid}$				0.64 $_{sid}$				1.55 $_{sid}$			
ICC	0.97				0.94				0.93				0.94				0.96			
N	20 $_{sid}$				20 $_{sid}$				20 $_{sid}$				20 $_{sid}$				20 $_{sid}$			
Observations	80				80				80				80				80			
Marginal R^2 / Conditional R^2	0.004 / 0.974				0.011 / 0.939				0.014 / 0.934				0.025 / 0.937				0.006 / 0.965			

Supplementary Table 7d. Linear mixed models for AON responses with togetherness x synchrony x gaze direction included as fixed effects in the model.

	**right IPS**				**right SPL**				**right STS**				**right FG**				**right lateral occipital**				**right dPMC**			
Predictors	*Estimates*	*CI*	*Statistic*	*p*	*Estimates*	*CI*	*Statistic*	*p*	*Estimates*	*CI*	*Statistic*	*p*	*Estimates*	*CI*	*Statistic*	*p*	*Estimates*	*CI*	*Statistic*	*p*	*Estimates*	*CI*	*Statistic*	*p*
(Intercept)	0.84	0.37, 1.30	3.55	**0.001**	0.19	-0.20, 0.57	0.98	0.330	1.29	0.91, 1.67	6.73	**<0.001**	1.84	1.43, 2.26	8.83	**<0.001**	3.33	2.56, 4.11	8.55	**<0.001**	0.47	0.12, 0.82	2.65	**0.010**
gaze.c	0.16	-0.04, 0.35	1.61	0.112	0.12	-0.07, 0.31	1.30	0.196	0.05	-0.13, 0.22	0.55	0.585	0.09	-0.14, 0.31	0.77	0.444	0.06	-0.23, 0.34	0.39	0.694	0.03	-0.13, 0.18	0.33	0.743
sync.c	0.07	-0.17, 0.31	0.60	0.552	0.01	-0.22, 0.24	0.13	0.899	-0.33	-0.54, -0.12	-3.09	**0.003**	-0.04	-0.31, 0.23	-0.30	0.763	-0.21	-0.56, 0.14	-1.20	0.233	-0.15	-0.33, 0.04	-1.56	0.122
sync.c:gaze.c	0.15	-0.19, 0.49	0.87	0.387	0.09	-0.25, 0.43	0.53	0.598	0.14	-0.17, 0.44	0.88	0.382	-0.03	-0.43, 0.36	-0.16	0.875	-0.07	-0.58, 0.44	-0.27	0.787	0.13	-0.14, 0.40	0.95	0.348
togetherness.c	-0.04	-0.18, 0.09	-0.62	0.535	0.01	-0.12, 0.15	0.22	0.828	0.12	-0.00, 0.24	1.99	0.051	0.10	-0.05, 0.26	1.33	0.187	0.15	-0.05, 0.35	1.46	0.149	0.08	-0.02, 0.19	1.53	0.131
togetherness.c:gaze.c	-0.06	-0.23, 0.11	-0.69	0.493	-0.08	-0.25, 0.08	-1.01	0.318	-0.08	-0.23, 0.07	-1.03	0.309	0.09	-0.11, 0.28	0.90	0.370	0.02	-0.23, 0.26	0.12	0.903	-0.08	-0.21, 0.06	-1.16	0.249
togetherness.c:sync.c	-0.14	-0.33, 0.04	-1.58	0.119	-0.08	-0.26, 0.10	-0.89	0.379	-0.17	-0.33, -0.01	-2.09	**0.040**	-0.03	-0.24, 0.17	-0.33	0.740	-0.10	-0.37, 0.16	-0.78	0.441	-0.20	-0.34, -0.05	-2.74	**0.008**
togetherness.c:sync.c:gaze.c	-0.16	-0.51, 0.18	-0.94	0.350	-0.07	-0.41, 0.27	-0.42	0.674	0.06	-0.25, 0.38	0.40	0.689	-0.21	-0.61, 0.19	-1.04	0.300	0.07	-0.44, 0.59	0.28	0.778	0.05	-0.22, 0.33	0.38	0.702
**Random Effects**																								
σ^2	0.05				0.05				0.04				0.07				0.11				0.03			
τ_{00}	1.07 $_{\text{sid}}$				0.70 $_{\text{sid}}$				0.70 $_{\text{sid}}$				0.82 $_{\text{sid}}$				2.96 $_{\text{sid}}$				0.60 $_{\text{sid}}$			
ICC	0.95				0.94				0.95				0.92				0.96				0.95			
N	20 $_{\text{sid}}$				20 $_{\text{sid}}$				20 $_{\text{sid}}$				20 $_{\text{sid}}$				20 $_{\text{sid}}$				20 $_{\text{sid}}$			
Observations	80				80				80				80				80				80			
Marginal R^2 / Conditional R^2	0.004 / 0.955				0.006 / 0.936				0.026 / 0.947				0.013 / 0.926				0.006 / 0.964				0.015 / 0.951			

Supplementary Table 8a. Linear mixed models for TOM responses with liking x synchrony x gaze direction included as fixed effects in the model.

	dMPFC				left STS				left TPJ				left TP			
Predictors	*Estimates*	*CI*	*Statistic*	*p*	*Estimates*	*CI*	*Statistic*	*p*	*Estimates*	*CI*	*Statistic*	*p*	*Estimates*	*CI*	*Statistic*	*p*
(Intercept)	-1.01	-1.50, -0.53	-4.17	**<0.001**	0.17	-0.28, 0.61	0.74	0.459	-0.07	-0.52, 0.38	-0.30	0.768	0.75	0.22, 1.27	2.82	**0.006**
gaze.c	0.18	-0.04, 0.40	1.61	0.113	0.07	-0.07, 0.20	0.98	0.331	0.20	0.06, 0.34	2.89	**0.005**	0.06	-0.11, 0.23	0.73	0.470
liking.c	-0.06	-0.21, 0.10	-0.70	0.489	0.03	-0.07, 0.12	0.56	0.579	-0.05	-0.15, 0.05	-0.99	0.326	-0.02	-0.14, 0.10	-0.34	0.734
liking.c:gaze.c	0.12	-0.12, 0.35	0.99	0.326	-0.00	-0.14, 0.14	-0.02	0.986	0.04	-0.11, 0.18	0.52	0.608	-0.09	-0.27, 0.09	-1.02	0.313
liking.c:sync.c	-0.14	-0.40, 0.12	-1.11	0.272	0.01	-0.15, 0.16	0.08	0.933	-0.12	-0.28, 0.04	-1.48	0.144	-0.03	-0.23, 0.16	-0.34	0.735
liking.c:sync.c:gaze.c	-0.32	-0.77, 0.13	-1.41	0.162	-0.20	-0.47, 0.07	-1.46	0.150	-0.30	-0.58, -0.02	-2.17	**0.034**	-0.33	-0.67, 0.01	-1.93	0.058
sync.c	0.26	0.00, 0.52	2.03	**0.046**	0.02	-0.13, 0.18	0.29	0.774	0.17	0.01, 0.33	2.07	**0.043**	0.06	-0.14, 0.26	0.58	0.561
sync.c:gaze.c	0.15	-0.30, 0.59	0.66	0.511	0.08	-0.19, 0.35	0.63	0.533	0.01	-0.26, 0.29	0.10	0.918	0.19	-0.15, 0.53	1.10	0.275
**Random Effects**																
σ^2	0.14				0.05				0.05				0.08			
τ_{00}	1.11 $_{sid}$				0.96 $_{sid}$				1.00 $_{sid}$				1.36 $_{sid}$			
ICC	0.89				0.95				0.95				0.94			
N	20 $_{sid}$				20 $_{sid}$				20 $_{sid}$				20 $_{sid}$			
Observations	80				80				80				80			
Marginal R^2 / Conditional R^2	0.019 / 0.891				0.003 / 0.950				0.011 / 0.951				0.004 / 0.945			

Supplementary Table 8b. Linear mixed models for TOM responses with liking x synchrony x gaze direction included as fixed effects in the model.

	**precuneus**				**right STS**				**right TPJ**				**right TP**				**vMPFC**			
Predictors	*Estimates*	*CI*	*Statistic*	*p*	*Estimates*	*CI*	*Statistic*	*p*	*Estimates*	*CI*	*Statistic*	*p*	*Estimates*	*CI*	*Statistic*	*p*	*Estimates*	*CI*	*Statistic*	*p*
(Intercept)	-0.93	-1.40, -0.45	-3.91	**<0.001**	0.52	0.17, 0.87	2.98	**0.004**	-0.01	-0.53, 0.52	-0.03	0.978	0.43	-0.07, 0.94	1.71	0.091	-2.06	-3.03, -1.09	-4.22	**<0.001**
gaze.c	0.16	-0.05, 0.37	1.50	0.138	0.15	0.02, 0.27	2.35	**0.022**	0.16	0.03, 0.29	2.49	**0.015**	0.03	-0.13, 0.18	0.36	0.722	0.19	-0.15, 0.53	1.12	0.268
liking.c	-0.07	-0.22, 0.08	-0.96	0.341	0.01	-0.08, 0.10	0.25	0.805	-0.07	-0.16, 0.02	-1.46	0.148	-0.00	-0.11, 0.11	-0.02	0.982	0.07	-0.17, 0.31	0.56	0.581
liking.c:gaze.c	0.05	-0.17, 0.27	0.43	0.667	0.08	-0.05, 0.21	1.18	0.241	0.05	-0.08, 0.18	0.76	0.452	-0.07	-0.23, 0.09	-0.84	0.404	0.12	-0.23, 0.47	0.67	0.505
liking.c:sync.c	-0.10	-0.35, 0.14	-0.84	0.403	-0.08	-0.23, 0.07	-1.09	0.280	0.05	-0.10, 0.20	0.64	0.522	-0.05	-0.23, 0.13	-0.51	0.612	0.26	-0.14, 0.65	1.29	0.200
liking.c:sync.c:gaze.c	-0.36	-0.78, 0.07	-1.66	0.101	-0.16	-0.41, 0.09	-1.24	0.218	-0.18	-0.44, 0.07	-1.42	0.159	-0.21	-0.52, 0.10	-1.33	0.188	-0.36	-1.04, 0.32	-1.05	0.296
sync.c	0.13	-0.12, 0.38	1.05	0.299	-0.01	-0.16, 0.13	-0.20	0.839	-0.04	-0.19, 0.10	-0.60	0.552	0.05	-0.14, 0.23	0.50	0.616	0.20	-0.19, 0.60	1.02	0.310
sync.c:gaze.c	0.02	-0.40, 0.45	0.10	0.917	-0.15	-0.40, 0.11	-1.16	0.251	-0.12	-0.38, 0.13	-0.95	0.346	0.01	-0.30, 0.32	0.04	0.965	-0.39	-1.06, 0.29	-1.14	0.259
**Random Effects**																				
σ^2	0.13				0.04				0.05				0.07				0.32			
τ_{00}	1.06_{sid}				0.59_{sid}				1.36_{sid}				1.24_{sid}				4.60_{sid}			
ICC	0.89				0.93				0.97				0.95				0.94			
N	20_{sid}				20_{sid}				20_{sid}				20_{sid}				20_{sid}			
Observations	80				80				80				80				80			
Marginal R^2 / Conditional R^2	0.009 / 0.895				0.011 / 0.932				0.008 / 0.968				0.003 / 0.949				0.008 / 0.936			

Supplementary Table 9a. Linear mixed models for TOM responses with togetherness x synchrony x gaze direction included as fixed effects in the model.

	dMPFC				left STS				left TPJ				left TP			
Predictors	*Estimates*	*CI*	*Statistic*	*p*	*Estimates*	*CI*	*Statistic*	*p*	*Estimates*	*CI*	*Statistic*	*p*	*Estimates*	*CI*	*Statistic*	*p*
(Intercept)	-1.05	-1.54, -0.55	-4.21	**<0.001**	0.20	-0.24, 0.65	0.92	0.361	-0.10	-0.55, 0.35	-0.45	0.656	0.76	0.22, 1.30	2.83	**0.006**
gaze.c	0.02	-0.30, 0.35	0.15	0.885	-0.04	-0.23, 0.16	-0.39	0.698	0.04	-0.17, 0.25	0.38	0.707	0.08	-0.16, 0.32	0.67	0.504
sync.c	-0.01	-0.40, 0.38	-0.04	0.970	-0.07	-0.31, 0.16	-0.62	0.538	-0.07	-0.32, 0.18	-0.58	0.566	0.00	-0.28, 0.29	0.03	0.979
sync.c:gaze.c	0.36	-0.21, 0.94	1.26	0.213	0.15	-0.19, 0.50	0.88	0.380	0.10	-0.27, 0.46	0.54	0.593	0.24	-0.18, 0.66	1.14	0.260
togetherness.c	0.13	-0.09, 0.36	1.17	0.247	0.08	-0.05, 0.22	1.20	0.235	0.11	-0.03, 0.26	1.56	0.122	0.05	-0.11, 0.22	0.64	0.524
togetherness.c:gaze.c	-0.10	-0.38, 0.19	-0.68	0.501	-0.02	-0.19, 0.15	-0.26	0.794	-0.06	-0.24, 0.12	-0.67	0.506	-0.11	-0.32, 0.09	-1.09	0.278
togetherness.c:sync.c	0.05	-0.25, 0.36	0.34	0.734	-0.09	-0.27, 0.09	-0.98	0.332	0.04	-0.15, 0.23	0.40	0.689	-0.02	-0.24, 0.20	-0.17	0.865
togetherness.c:sync.c:gaze.c	-0.05	-0.64, 0.53	-0.17	0.864	0.02	-0.33, 0.37	0.12	0.906	0.00	-0.37, 0.38	0.03	0.979	-0.43	-0.86, -0.01	-2.03	**0.047**
**Random Effects**																
σ^2	0.14				0.05				0.06				0.08			
τ_{00}	1.13 $_{sid}$				0.95 $_{sid}$				0.97 $_{sid}$				1.39 $_{sid}$			
ICC	0.89				0.95				0.94				0.95			
N	20 $_{sid}$				20 $_{sid}$				20 $_{sid}$				20 $_{sid}$			
Observations	80				80				80				80			
Marginal R^2 / Conditional R^2	0.018 / 0.889				0.005 / 0.950				0.010 / 0.945				0.007 / 0.948			

Supplementary Table 9b. Linear mixed models for TOM responses with togetherness x synchrony x gaze direction included as fixed effects in the model.

	precuneus				right STS				right TPJ				right TP				vMPFC			
Predictors	*Estimates*	*CI*	*Statistic*	*p*	*Estimates*	*CI*	*Statistic*	*p*	*Estimates*	*CI*	*Statistic*	*p*	*Estimates*	*CI*	*Statistic*	*p*	*Estimates*	*CI*	*Statistic*	*p*
(Intercept)	-0.93	-1.41, -0.46	-3.91	**<0.001**	0.54	0.19, 0.89	3.05	**0.003**	0.07	-0.46, 0.60	0.28	0.782	0.36	-0.14, 0.87	1.45	0.151	-1.92	-2.91, -0.93	-3.87	**<0.001**
gaze.c	-0.09	-0.40, 0.22	-0.58	0.561	0.08	-0.10, 0.26	0.90	0.374	-0.07	-0.25, 0.10	-0.83	0.411	-0.03	-0.24, 0.18	-0.27	0.791	-0.09	-0.57, 0.39	-0.36	0.722
sync.c	-0.23	-0.60, 0.15	-1.22	0.227	-0.15	-0.37, 0.07	-1.36	0.178	-0.32	-0.54, -0.10	-2.96	**0.004**	-0.09	-0.35, 0.17	-0.70	0.487	-0.15	-0.73, 0.43	-0.52	0.607
sync.c:gaze.c	0.12	-0.43, 0.68	0.45	0.654	0.05	-0.27, 0.37	0.33	0.740	0.15	-0.16, 0.47	0.98	0.332	-0.14	-0.52, 0.23	-0.76	0.449	0.13	-0.72, 0.98	0.31	0.757
togetherness.c	0.16	-0.05, 0.38	1.52	0.134	0.10	-0.02, 0.23	1.64	0.106	0.10	-0.02, 0.22	1.59	0.115	0.11	-0.04, 0.26	1.46	0.148	0.29	-0.05, 0.62	1.73	0.089
togetherness.c:gaze.c	-0.03	-0.30, 0.24	-0.21	0.833	-0.07	-0.22, 0.09	-0.87	0.386	-0.08	-0.23, 0.08	-1.02	0.313	-0.03	-0.21, 0.16	-0.31	0.758	-0.23	-0.64, 0.19	-1.09	0.281
togetherness.c:sync.c	-0.04	-0.33, 0.26	-0.25	0.803	-0.07	-0.24, 0.10	-0.83	0.407	-0.14	-0.31, 0.02	-1.73	0.088	0.17	-0.03, 0.37	1.65	0.103	-0.05	-0.50, 0.40	-0.21	0.836
togetherness.c:sync.c:gaze.c	0.12	-0.44, 0.68	0.43	0.666	-0.08	-0.40, 0.24	-0.49	0.627	0.30	-0.02, 0.62	1.88	0.064	-0.19	-0.57, 0.19	-0.99	0.326	0.03	-0.84, 0.89	0.06	0.953
**Random Effects**																				
σ^2	0.13				0.04				0.04				0.06				0.31			
τ_{00}	1.04 sid				0.59 sid				1.37 sid				1.21 sid				4.67 sid			
ICC	0.89				0.93				0.97				0.95				0.94			
N	20 sid				20 sid				20 sid				20 sid				20 sid			
Observations	80				80				80				80				80			
Marginal R^2 / Conditional R^2	0.010 / 0.889				0.015 / 0.932				0.010 / 0.970				0.009 / 0.953				0.012 / 0.938			

Supplementary Table 10. MNI coordinates for the action observation and theory of mind network ROIs.

	Action Observation Network			
	**ROI**	**x**	**y**	**z**
**1**	L IFG	− 50	9	30
**2**	L dPMC	− 26	− 4	56
**3**	L SMA	− 2	18	50
**4**	L SI	− 34	− 44	52
**5**	L IPL	− 60	− 24	36
**6**	L STS	− 54	− 50	8
**7**	L lat occipital	− 46	− 72	2
**8**	L Fusiform	− 44	− 56	− 18
**9**	R IFG	52	12	26
**10**	R IFG 2	56	30	− 2
**11**	R dPMC	34	− 2	54
**12**	R SMA	4	12	58
**13**	R SI	60	− 20	40
**14**	R IPL	44	− 34	44
**15**	R SPL	22	− 62	64
**16**	R IPS	30	− 54	48
**17**	R STS	56	− 40	4
**18**	R lat occipital	52	− 64	0
**19**	R Fusiform	44	− 54	− 18

	Theory of Mind Network			
	**ROI**	**x**	**y**	**z**
**1**	Precuneus	0	-52	34
**2**	dmPFC	-6	54	22
**3**	vmPFC	4	46	-16
**4**	R TPJ	50	-54	22
**5**	R STS	50	-18	-12
**6**	R TP	52	4	-34
**7**	L TPJ	-50	-60	24
**8**	L STS	-52	-2	-22
**9**	L TP	-52	6	-32

Supplementary Table 11. Demographic information for the behavioural and fMRI experiments.

**Experiment**		**Variable**	**N**	**Mean**	**SD**
fMRI	2012	Age	20	22.65	2.43
	2012	Dance experience	20	6.05	3.33
Behavioural	2012	Age	48	20.56	3.18
	2012	Dance experience	49	8.69	4.24
	2023	Age	57	28.91	7.95
	2023	Dance experience	57	8.37	4.24

Appendix A. Dance experience questionnaire scoring

1) During the last 4 years or so, how often per year (on average) do you go to see any kind o live performance that may have some kind of dance in it?

0 1-5 more than 5

2) How many dance films/documentaries did you watch during the last year?

3) How frequently do you dance at parties or clubs (number of nights/month)?

4) How good of a dancer do you think you are?

really bad bad intermediate good very good

5) *Have you ever taken any kind of dance class?* ***YES*** **NO**

6) In case you answered the former question with yes, please fill in the following questions for each dance style you took lessons in

a) which kind of dance lessons did you take?

b) when did you take them (for example, recently or 2 years ago)

c) how long did you take them (for example, for 2 years)

7) If you have any feedback on this study, please feel free to write it below:

Scoring:

For questions 1 and 2 a response of 0, 1-5 or more than 5 became 0, 1, and 2 respectively. For question 3 a response of 0, 1-5, 6-10 or more than 10 became 0, 1, 2, and 3 respectively. For question 4 really bad = 0, bad = 1, intermediate = 3, good = 4, really good = 5. Question 5, no = 0 and yes = 1.

Question 6a the number is just how many different types of dance the participant has taken classes in.

For question 6b the participant got a score of:

0 = they stopped taking them > 5 years ago

1 = they stopped taking them 1-5 years ago

2 = they are taking them presently/ stopped < a year ago.

And finally, for 6c they got a score of:

0 = if they took lessons for < 1 year.

1 = if they took lessons for 1-5 years.

2 = if they took lessons for > 6 years.

We then totalled their scores to give one number.
